# A Comprehensive Analysis of the Effects of Key Mitophagy Genes on the Progression and Prognosis of Lung Adenocarcinoma

**DOI:** 10.3390/cancers15010057

**Published:** 2022-12-22

**Authors:** Dongjun Dai, Lihong Liu, Yinglu Guo, Yongjie Shui, Qichun Wei

**Affiliations:** Department of Radiation Oncology, The Second Affiliated Hospital, Zhejiang University School of Medicine, Hangzhou 310009, China

**Keywords:** lung adenocarcinoma, mitophagy, TCGA, GEO, immunotherapy, prognosis

## Abstract

**Simple Summary:**

Dysfunction and dysregulation of mitochondrial dynamics are implicated in tumorigenesis. To avoid mitochondrial dysfunction, a quality control mechanism called mitophagy is developed in cells. A series of mitophagy-related genes were identified to be associated with lung cancer. However, there was no comprehensive genetic and transcriptional analysis of all key mitophagy genes in lung adenocarcinoma (LUAD) progression, prognosis and therapeutics. Here, we performed a comprehensive analysis of the gene expression, copy number variation and mutation of key mitophagy genes in the tumor progression of LUAD. The clustering analysis identified two groups of LUAD with a significantly different prognosis. Further analyses of gene mutation, genome profile, immune cell infiltration and drug sensitivity were performed between these two groups. We also constructed a mitophagy related signature that could predict the prognosis of LUAD, which was validated in external database. This study was valuable for LUAD prognostic prediction and treatment decision.

**Abstract:**

The aim of our study was to perform a comprehensive analysis of the gene expression, copy number variation (CNV) and mutation of key mitophagy genes in the progression and prognosis of lung adenocarcinoma (LUAD). We obtained the data from The Cancer Genome Atlas (TCGA). Clustering analysis was performed to stratify the mitophagy related groups. The least absolute shrinkage and selection operator (LASSO) based cox model was used to select hub survival genes. An independent validation cohort was retrieved from Gene Expression Omnibus database. We found 24 out of 27 mitophagy genes were aberrantly expressed between tumor and normal samples. CNV gains were associated with higher expression of mitophagy genes in 23 of 27 mitophagy genes. The clustering analysis identified high and low risk mitophagy groups with distinct survival differences. The high risk mitophagy groups had higher tumor mutation burden, stemness phenotype, total CNVs and lower CD4+ T cells infiltration. Drugs targeted to high risk mitophagy groups were identified including the PI3K/AKT/mTOR inhibitor, HDAC inhibitor and chemotherapy agents such as cisplatin and gemcitabine. In addition, the differentially expressed genes (DEGs) were identified between mitophagy groups. Further univariate Cox analysis of each DEG and subsequent LASSO-based Cox model revealed a mitophagy-related prognostic signature. The risk score model of this signature showed a strong ability to predict the overall survival of LUAD patients in training and validation datasets. In conclusion, the mitophagy genes played an important role in the progression and prognosis of LUAD, which might provide useful information for the treatment of LUAD.

## 1. Introduction

Lung cancer is the leading cause of cancer related death worldwide [1]. In the United States, the 5-year lung cancer survival rate was about 20% [2]. Nearly 80–85% lung cancer cases are non-small cell lung cancer (NSCLC) [3,4]. Lung adenocarcinoma (LUAD), accounts for nearly 40% of the NSCLC, is the most common type of lung cancer that occurs in never smokers and with more heterogenous genetic alterations [5]. Recently, with the increased understanding of the genetic basis, the development of immunotherapy and targeted therapy agents dramatically improved the prognosis of NSCLC patients, especially for LUAD [3,6].

Mitochondria provides basic materials for tumor anabolism, which is engaged in several biological processes such as tumor cell redox, transcription regulation, cell death control and host immune system [7]. Dysfunction and dysregulation of mitochondrial dynamics are implicated in tumorigenesis [8]. To avoid mitochondrial dysfunction, a quality control mechanism called mitophagy is developed in cells, which is a specific autophagy to clear damaged mitochondria [7]. Defects of mitophagy are associated with impaired mitochondrial function in multiple cancers, which promote oncogenesis and affects anti-cancer therapies [8].

Recently, a series of mitophagy related genes were identified to be associated with lung cancer [9]. For example, PINK1 depletion altered energetic metabolism and confers sensitivity to agents that inhibited glycolysis in NSCLC [10]. Depletion of ATG5 reduced tumor growth in RAS mutation lung cancer cell line [11]. Pro-apoptotic compound fluorizoline was identified to inhibit PRKN-dependent mitophagy and lower the viability of lung cancer cell line [12]. However, there was no comprehensive genetic and transcriptional analysis of all key mitophagy genes in LUAD progression, prognosis and therapeutics.

Here, we performed a comprehensive analysis of the gene expression, copy number variation and mutation of key mitophagy genes in the tumor progression of LUAD by using the data from The Cancer Genome Atlas (TCGA). The clustering analysis identified two groups of LUAD with a significantly different prognosis. Further analyses of gene mutation, genome profile, immune cell infiltration and drug sensitivity were performed between these two groups. We also constructed a mitophagy related signature that could predict the prognosis of LUAD, which was validated in Gene Expression Omnibus (GEO) database.

## 2. Materials and Methods

### 2.1. Data Collection

The transcription and copy number variation data of TCGA-LUAD was obtained from Xena database [13]. The mutation data was obtained by R package “TCGAmutation”. The transcriptional counts data was normalized by “TMM” method [14] and transformed by “voom” method from “limma” R package [15]. The key mitophagy genes were selected by referring to a previous study which identified the mitophagy genes from Reactome database [16]. The selected 28 mitophagy genes comprised ATG12, ATG5, CSNK2A1, CSNK2A2, CSNK2B, FUNDC1, MAP1LC3A, MAP1LC3B, MFN1, MFN2, MTERF3, PGAM5, PINK1, PRKN, RPS27A, SQSTM1, SRC, TOMM20, TOMM22, TOMM40, TOMM5, TOMM6, TOMM7, UBA52, UBB, UBC, ULK1 and VDAC1. We only included the 27 mitophagy genes in the analysis since we discovered that the TOMM6 had 0 read counts in all samples.

The LUAD patients with RNA count data were randomized and stratified into a training group (n = 332) and a validation group (n = 184). The prognostic analysis was performed in the training group and validated by the TCGA-LUAD validation group, the whole TCGA-LUAD group and an external dataset from GEO database (GSE72094) [17].

The R package “ConsensusClusterPlus” was used to stratify the mitophagy related clusters [18]. The parameter settings were as follows: reps = 1000, pItem = 0.8, pFeature = 1, and distance = Euclidean. The stratified mitophagy groups were then to be used in next analyses.

### 2.2. The Association between Tumor Mutation Burden (TMB) and Mitophagy Groups

TMB was defined as the number of non-synonymous alterations per megabase (Mb) of the genome, and we estimated the size of an exome to be 38 Mb [19]. The Wilcoxon analysis was used to evaluate the difference between mitophagy groups.

### 2.3. Evaluation of Cell Stemness in Mitophagy Groups

The mRNAsi score for the TCGA-LUAD samples was acquired from Malta et al. [20]. The mRNAsi score is a gene expression-based stemness index for assessing cancer cell dedifferentiation, which ranges from 0 to 1. The difference of mRNAsi score between mitophagy groups was assessed by Wilcoxon analysis.

### 2.4. Assessment of Immune Checkpoint Gene Expression in Mitophagy Groups

Violin plots were used to describe the expression levels of 8 immune-checkpoint genes in the two mitophagy groups. The immune-checkpoint genes comprised CD274, cytotoxic T-lymphocyte associated protein 4 (CTLA4), hepatitis A virus cellular receptor 2 (HAVCR2), lymphocyte activating 3 (LAG3), programmed cell death 1 (PDCD1), programmed cell death 1 ligand 2 (PDCD1LG2), sialic acid binding Ig like lectin 15 (SIGLEC15), and T cell immunoreceptor with Ig and ITIM domains (TIGIT). The difference of immune-checkpoint gene expression between mitophagy groups was assessed by Wilcoxon analysis.

### 2.5. Assessment of Tumor Immune Cell Infiltration in Mitophagy Groups

The ESTIMATE algorithm was used to compute the immune and stromal scores by R package “estimate”. We also estimated the immune cell fractions between mitophagy groups by R package “immunedeconv”, which included xCell, quanTIseq, EPIC and TIMER algorithms. The Fragments Per Kilobase Million (FPKM) data were downloaded from Xena database and transformed to Transcripts Per Million (TPM) value, which was utilized as input for R package "immunedeconv".

### 2.6. Differentially Expressed Genes (DEGs) Screening

The DEGs screening between mitophagy groups was performed by R package “limma”. DEGs were defined as genes having a fold change of more than 2 and a p-value less than 0.05. The volcano plot was created to visualize the DEGs results. Further analyses were conducted using the calculated DEGs.

### 2.7. Functional Analyses of DEGs

The functional analyses of DEGs included the gene ontology (GO) analysis, Kyoto Encyclopedia of Genes and Genomes (KEGG) analysis, and Gene Set Enrichment Analysis (GSEA). These analyses were performed by R package “clusterProfiler”.

### 2.8. The Prognostic Model Construction

The univariate Cox analysis was applied to each DEG. The cutoff point was defined as the median gene expression value. The survival related DEGs were defined as genes with a *p* value less than 0.05. The selected survival related DEGs were then analyzed by a least absolute shrinkage and selection operator (LASSO) analysis to identify hub survival genes (10-fold cross-validation). A risk score prognostic model based on hub survival genes was constructed by using the following formula: (βi × Expi) (i was the number of hub survival-related genes). The KM method was applied to the risk score for the overall survival (OS) of LUAD patients. The area under the receiver operating characteristic curve (AUC) plots were drawn to estimate the reliability of the risk score. The validation was performed in TCGA-LUAD testing cohort, whole TCGA-LUAD cohort and an external dataset from GEO database (GSE72094).

A nomogram was constructed by R package “rms” to predict the 1-year, 2-year, and 3-year OS of patients with LUAD. The validation was performed internally by calibration curve.

### 2.9. Drug Identification Analysis

We further explored the potential drugs that might target to high risk mitophagy group and assessed the drug response difference between mitophagy groups. The drug identification was performed by utilizing Connectivity Map (CMap) analysis [21]. The CMap analysis was conducted by R package “DrInsight” [22]. The drug-response prediction was assessed using R package “oncoPredict” [23]. The sensitivity scores were computed to the drugs for LUAD patients, the lower score presented sensitive status and the high score presented resistant status to a specific drug.

### 2.10. Statistical Analyses

All the statistical analyses were performed by R-4.1.3. The heatmap was plotted by R package “pheatmap”. The KM method was performed by R package “survminer”. LASSO analysis was performed by R package “glmnet”. AUC analysis was conducted by R package “timeROC”. The KM plots, violin plots, volcano plots were all drawn by R package “ggplot2”. For comparisons between two groups, Wilcoxon analysis was performed.

## 3. Results

### 3.1. The Landscape of Mitophagy Genes in LUAD

**Figure 1:**
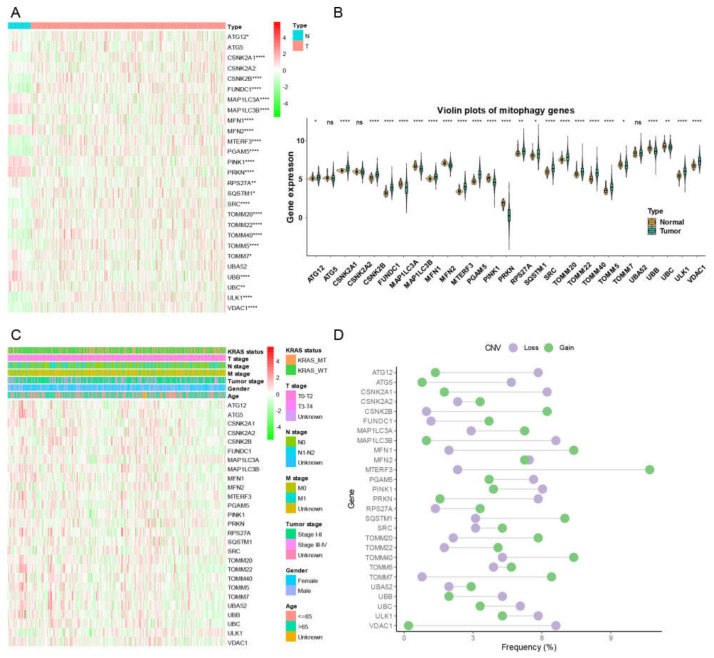
The landscape of gene expression and CNV of mitophagy genes in LUAD. (**A**) the heatmap of the gene expression level of mitophagy genes between normal (N) and tumor (T) tissues of LUAD in TCGA. (**B**) the violin plots which presented the expression of each mitophagy gene between normal and cancer tissues of LUAD in TCGA. (**C**) the expression of mitophagy genes in LUAD with clinicopathological features that comprises age, gender, T stage, M stage, N stage, tumor stage, and KRAS status. (**D**) the CNV distribution of mitophagy genes in TCGA database. The asterisks represented the statistical *p* value (* *p* < 0.05; ** *p* < 0.01; **** *p* < 0.0001, “ns” represents *p* value over 0.05).

**Figure 2:**
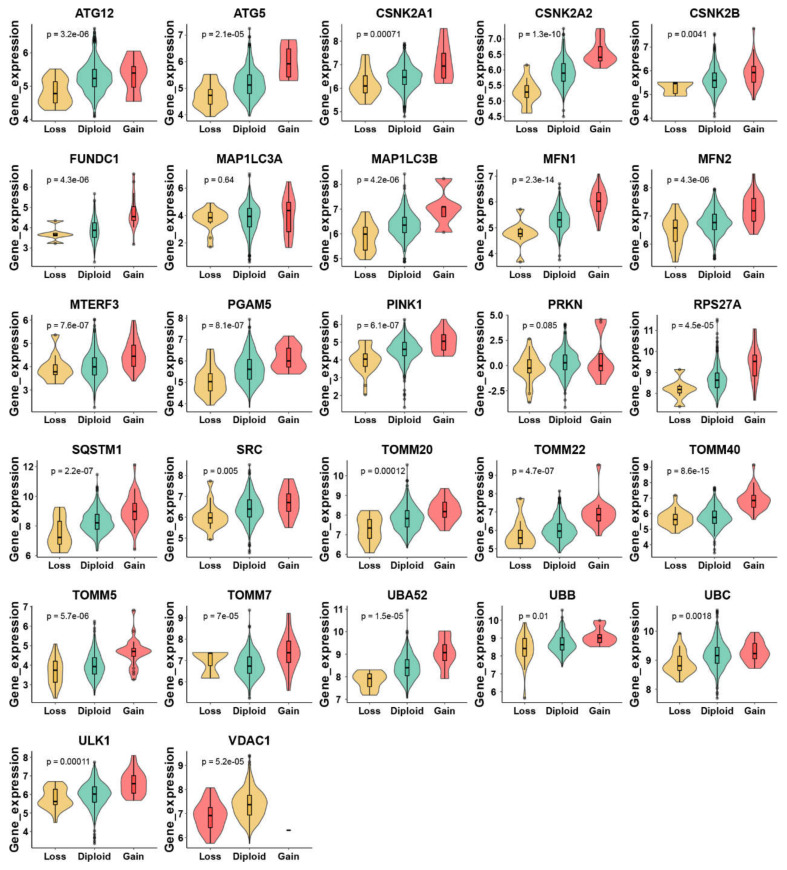
Relationship between CNV and mitophagy genes expression in LUAD by violin plots. The “e” in the *p* value represents E-notation. The E-notation is written as *m*En, which is equal to the scientific notation that is written as *m* × 10^n^.

There were 555 LUAD samples with read counts data collected from TCGA. We observed that ATG12, CSNK2A1, CSNK2B, FUNDC1, MFN1, MTERF3, PGAM5, RPS27A, SQSTM1, SRC, TOMM20, TOMM22, TOMM40, TOMM5, ULK1, VDAC1 were upregulated in tumor tissue while the MAP1LC3A, MAP1LC3B, MFN2, PINK1, PRKN, TOMM7, UBB, UBC were downregulated in tumor tissue (Figure 1A,B). A heatmap was constructed to show the relationships between mitophagy genes and the LUAD clinical factors that comprised age, gender, T stage, M stage, N stage, tumor stage, and KRAS status (Figure 1C). The details of the copy number alterations of mitophagy genes were listed in Figure 1D and Table S1. The MAP1LC3B and VDAC1 showed a higher proportion of loss in CNVs of LUAD patients while the MTERF3, MFN1, TOMM7 and CSNK2B showed a higher proportion of gain in CNVs of LUAD patients (Figure 1D, the difference was wider than 5%). We discovered that 23 out of 27 mitophagy genes’ CNV increases had a very strong correlation with higher gene expression (Except for MAP1LC3A, PRKN, TOMM7 and VDAC1, Figure 2). Mitophagy regulators were rarely mutated; the frequency was 11.6%. The mutation rate of each mitophagy gene ranged from 0.2% to 2%, with ULK1 and UBC having the highest mutation rate (Figure S1, Table S2).

### 3.2. Associations between Mitophagy Genes Expression and Clinicopathological Features

**Figure 3:**
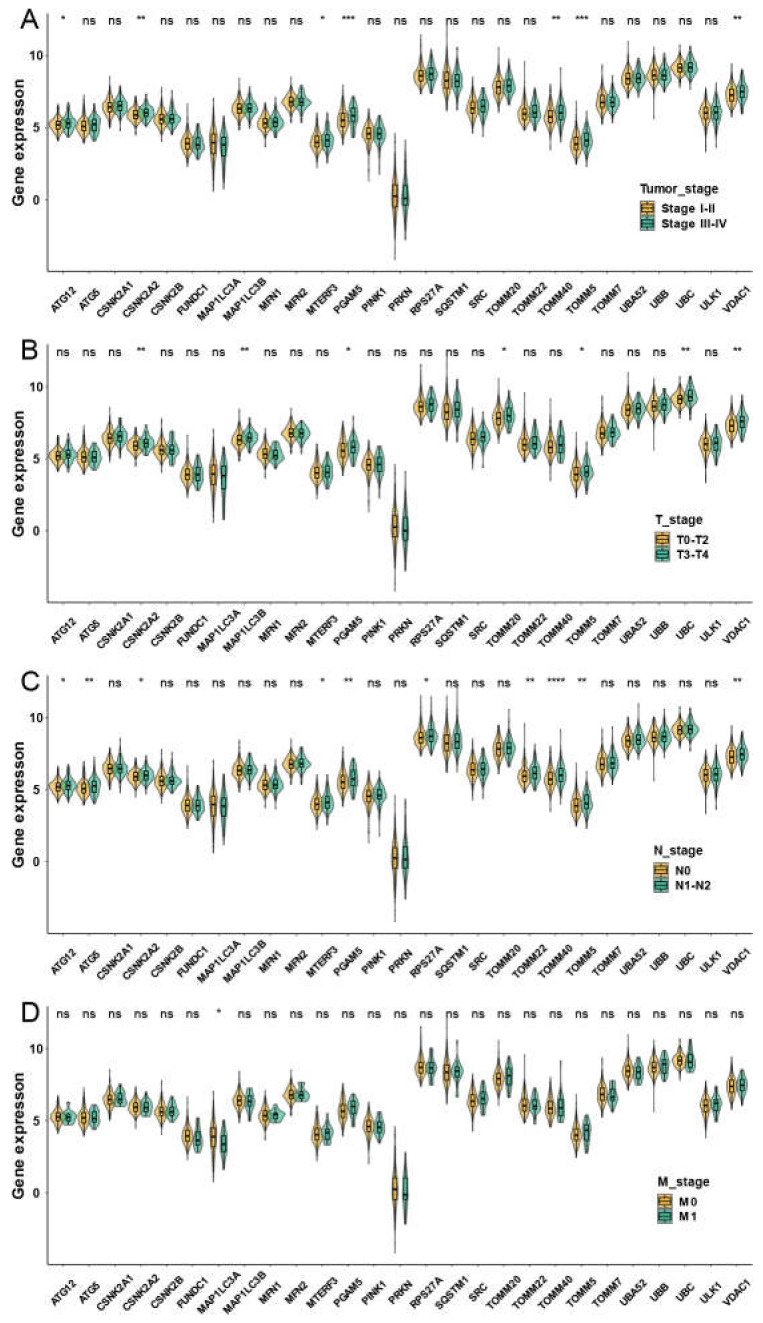
The correlation between the expression of mitophagy genes and the TNM stage in the TCGA LUAD cohort. The expression of mitophagy genes in different tumor stages (**A**), T stage (**B**), T stage (**C**) and M stage (**D**). The asterisks represented the statistical *p* value (* *p* < 0.05; ** *p* < 0.01; *** *p* < 0.001; **** *p* < 0.0001, “ns” represents *p* value over 0.05).

**Figure 4:**
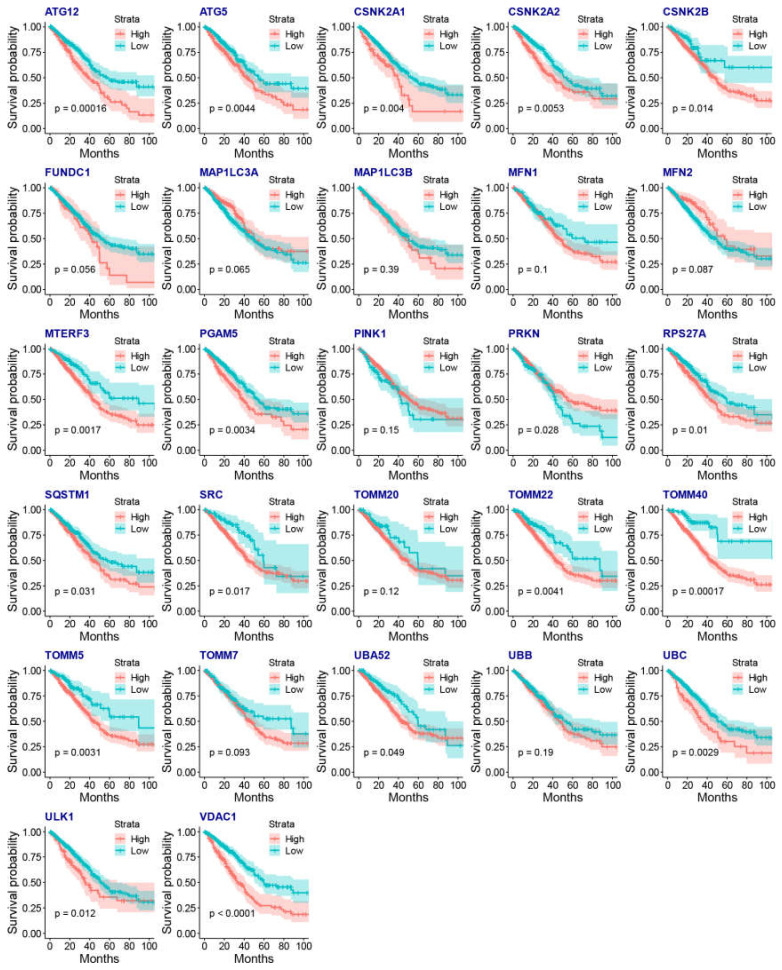
The association between the gene expression of mitophagy genes and the OS of LUAD by KM plots.

We then evaluated associations between mitophagy gene expression levels and clinicopathological features in LUAD. We found that the ATG12, CSNK2A2, MTERF3, PGAM5, TOMM40, TOMM5 and VDAC1 were higher in stage III-IV LUAD than the stage I-II LUAD (Figure 3A). We observed that the mitophagy gene might be associated with the tumor stage locally and lymphatic metastasis but not with distant metastasis in LUAD (Figure 3B–D). The survival analysis showed that higher expression of ATG12, ATG5, CSKN2A2, CSKN2A2, CSNK2B, MTERF3, PGAM5, RPS27A, SQSTM1, SRC, TOMM20, TOMM40, TOMM5, UBA52, UBC, ULK1 and VDAC1 were associated with inferior OS of LUAD, while the higher expression of PRKN was associated with favorable OS of LUAD (Figure 4).

### 3.3. Description of Mitophagy Subtypes in LUAD Patients

**Figure 5:**
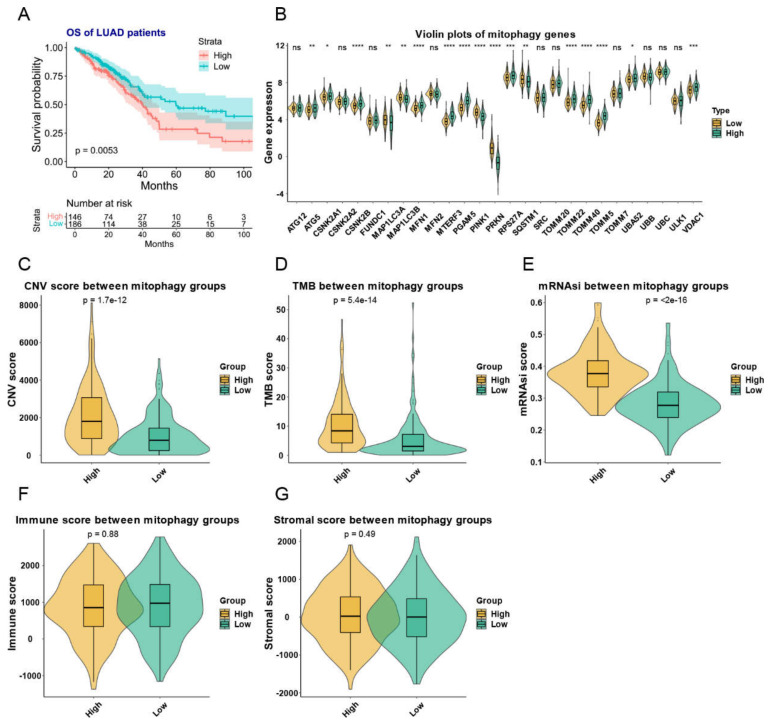
The mitophagy clusters and their relationship with TMB, total CNVs, stem cell phenotype and immune and stromal scores in LUAD. (**A**) the KM plot of the different mitophagy clusters. (**B**) The expression pattern of mitophagy genes between the two mitophagy groups. The asterisks represented the statistical *p* value (^ns^ *p* > 0.05; * *p* < 0.05; ** *p* < 0.01; *** *p* < 0.001; **** *p* < 0.0001). (**C**–**G**) the violin plots presented the association between mitophagy groups and TMB (**C**), total CNVs (**D**), stem cell phenotype (**E**) and immune (**F**) and stromal scores (**G**) in TCGA-LUAD cohort. The “e” in the *p* value of Figure (**C**–**E**) represents E-notation. The E-notation is written as mEn, which is equal to the scientific notation that is written as m × 10n.

For the purpose of constructing a prognostic model in LUAD patients, we stratified the LUAD patients with RNA counts data into a training group (n = 332) and a validation group (n = 165). A clustering analysis of the training group produced two groups with similar patterns of mitophagy gene expression. We discovered a significant prognostic difference between these two mitophagy groups (Figure 5A). We therefore named the two groups as high and low risk mitophagy groups. The detailed expression pattern of mitophagy genes were listed in Figure 5B. To be noted, there were 10 genes upregulated in high risk mitophagy group and in tumor tissues (CSKN2A1, CSKN2B, MFN1, MTERF3, PGAM5, RPS27A, TOMM22, TOMM40, TOMM5 and VDAC1, Figure 1A,B and Figure 5B) and 4 genes downregulated in high risk mitophagy group and the tumor tissues (MAP1LC3A, MAP1LC3B, PINK1 and PRKN, Figure 1A,B and Figure 5B).

**Figure 6:**
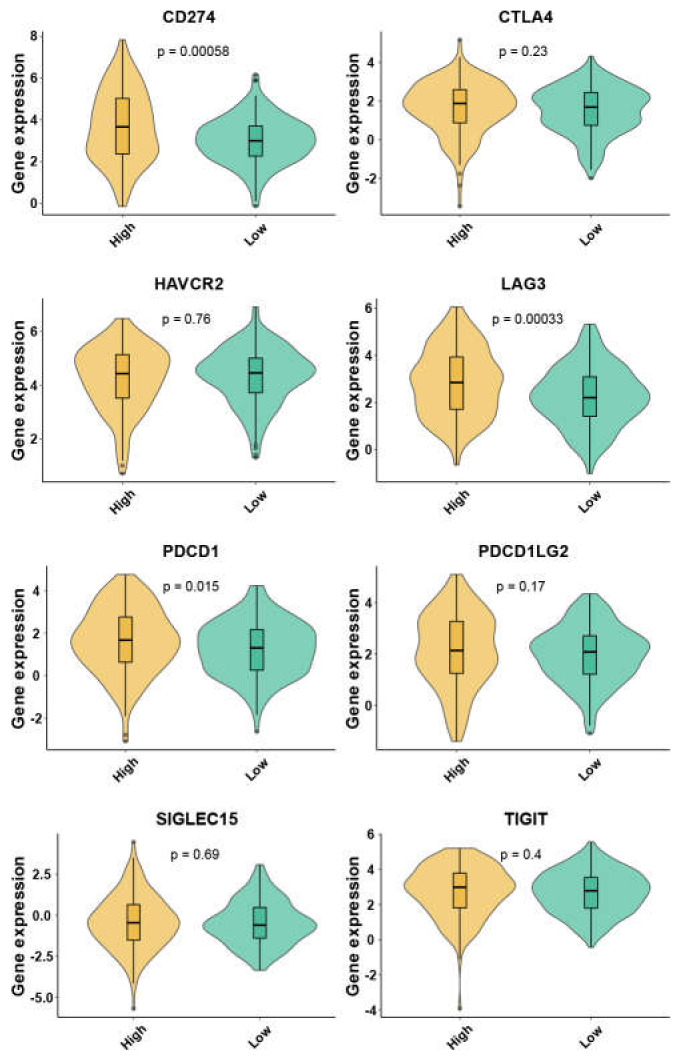
The expression of eight immune checkpoint family genes between two mitophagy groups by violin plots.

**Figure 7:**
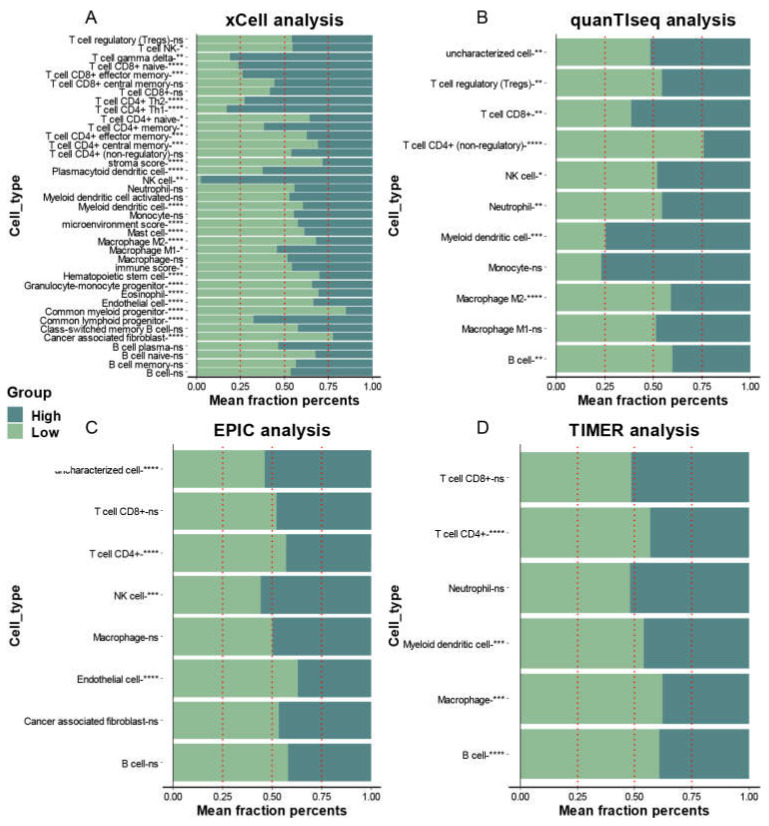
The association between infiltration level of immune cells and the mitophagy groups in LUAD. The xCell analysis (**A**), quanTIseq (**B**), EPIC (**C**) and TIMER (**D**) analyses of the two mitophagy groups. The x-axis is the mean cell fraction of a specific tumor immune contexture. The asterisks represented the statistical *p* value (* *p* < 0.05; ** *p* < 0.01; *** *p* < 0.001; **** *p* < 0.0001).

These two mitophagy groups were compared in a number of ways. We discovered that compared to the low-risk group, the high risk mitophagy group had higher total CNVs, mRNAsi scores, and TMB (Figure 5C–E). There was no association between mitophagy groups and ESTIMATE-immune score and stromal score (Figure 5F,G). Further we estimated the association between mitophagy groups and eight immune-checkpoint genes. We found that the CD274, LAG3 and PDCD1 were significantly higher in the high-risk mitophagy group (Figure 6). Moreover, we analyzed the immune cell fractions between two mitophagy groups, we found that the CD4+ T cell was lower in high risk mitophagy group by four different algorithms (Figure 7A–D).

### 3.4. The association between Mitophagy Groups and the Drug Response

We next try to explore the association between mitophagy groups and the drug response. The CMap analysis showed that histone deacetylases (HDAC) inhibitors trichostatin A and vorinostat, PI3K-AKT-mTOR pathway inhibitors LY-294002, sirolimus and wortmannin were potential drugs that targeted for high risk mitophagy group (Table 1, *p*-value < 0.05, FDR < 0.1). The “oncoPredict” package showed that the high risk mitophagy group had a lower sensitive score than low risk mitophagy group in a series of drugs (Table 1). To be noted, compared to low risk mitophagy group, the high risk mitophagy was more sensitive to cisplatin and gemcitabine, which were often used in the treatment of LUAD.

### 3.5. The DEGs Screening between Mitophagy Groups and the Related Functional Analyses

**Figure 8:**
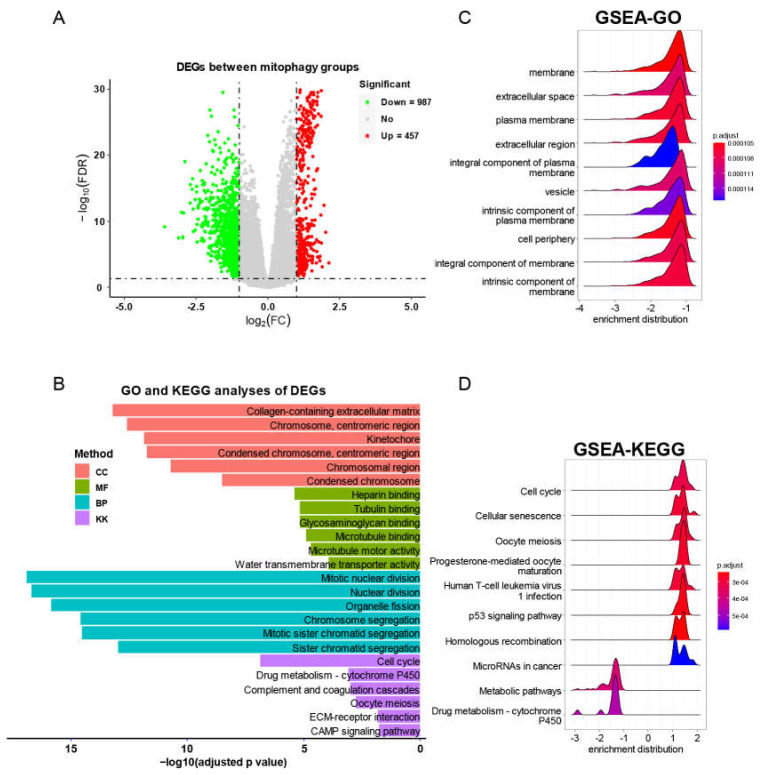
The DEG identification and related functional analyses. (**A**) The volcano plot of DEGs between mitophagy groups. (**B**) The GO and KEGG analyses of DEGs between mitophagy groups. (**C**,**D**) The GSEA of DEGs by GO (**C**) and KEGG (**D**) category.

We then screened the DEGs between mitophagy groups. As shown by volcano plot (Figure 8A), there were 457 upregulated genes and 987 downregulated genes (Table S3). We subsequently used these DEGs to perform functional analyses. The GO and KGEE analysis showed that nuclear division, chromosome segregation and cell cycle were associated with mitophagy (Figure 8B). The GSEA analysis identified a series of cancer associated pathways that were upregulated in high risk mitophagy group, such as cell cycle, cellular senescence, oocyte meiosis and p53 signaling pathways (Figure 8C,D). 

### 3.6. The Prognostic Signature Identification and Prognostic Model Construction

**Figure 9:**
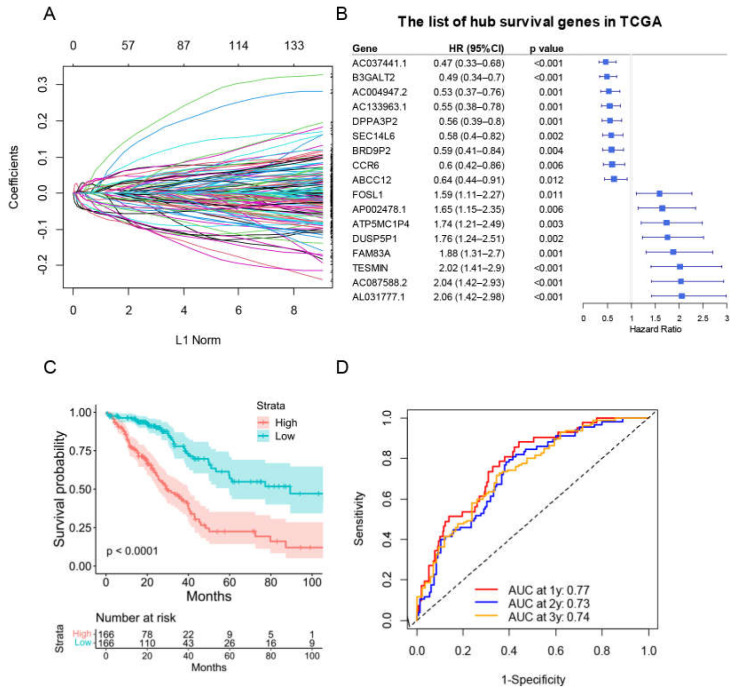
The construction of risk score model for the OS of LUAD patients in training group. (**A**) Selection of the mitophagy-related hub survival genes by LASSO Cox analysis. (**B**) The forest plot containing the selected hub survival genes in TCGA database. (**C**) The KM plot of the high or low risk score LUAD patients in TCGA. (**D**) The AUC plots of risk score to predict the 1-, 2-, and 3-year OS of LUAD in TCGA training cohort.

We next explored the potential mitophagy-related prognostic signature. We started by performing univariate Cox analysis in each mitophagy related DEG. We discovered 235 genes linked to an inferior OS of LUAD and 309 genes linked to a favorable OS of LUAD (Table S4). The selected survival related genes were then put into a LASSO-based Cox analysis. There were 17 genes (*CCR6, TESMIN, ABCC12, FAM83A, B3GALT2, FOSL1, DUSP5P1, DPPA3P2, SEC14L6, AL031777.1, ATP5MC1P4, AC133963.1, AC037441.1, BRD9P2, AP002478.1, AC087588.2* and *AC004947.2*) finally selected as hub survival genes, as they had nonzero coefficient values by the LASSO analysis (Figure 9A and Table S5). The detailed prognostic information of each hub gene was shown in a forest plot (Figure 9B).

**Figure 10:**
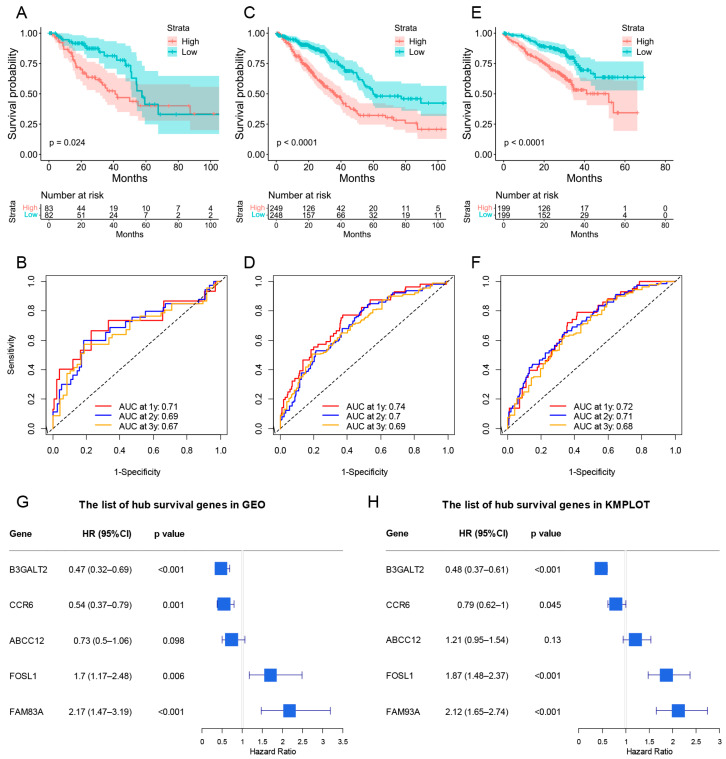
The validation of risk score model for predicting OS of LUAD. (**A**–**F**) The validations of KM plot and AUC plot were constructed from the TCGA-LUAD validation group (**A**,**B**), total TCGA-LUAD group (**C**,**D**), and GEO-LUAD group (**E**,**F**). (**G**,**H**) The forest plot containing the hub genes in GEO database and KMPLOT database (B3GALT2, CCR6, ABCC12, FOSL1, and FAM83A).

**Figure 11:**
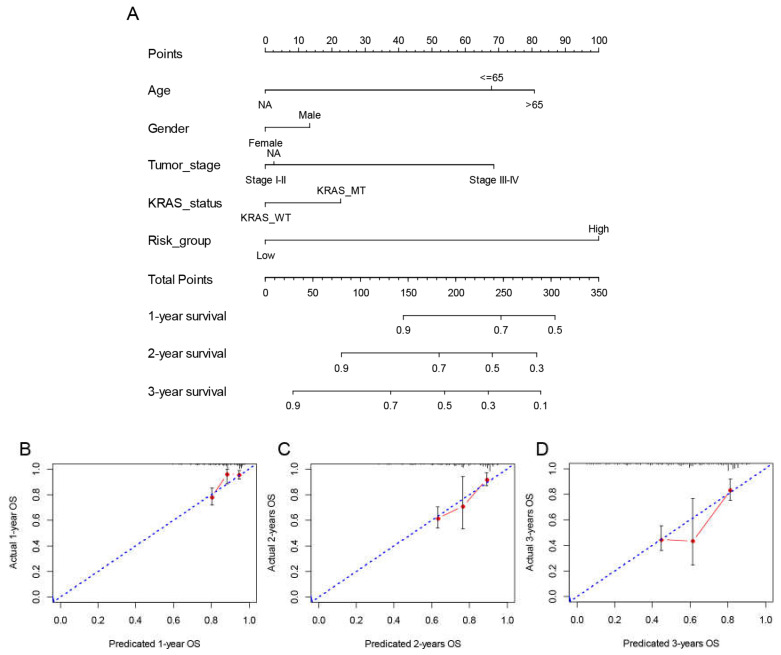
Construction and validation of the nomogram to predict OS in the TCGA LUAD cohort. (**A**) The multivariate Cox model-based nomogram of LUAD patients in TCGA training group. (**B**–**D**) the calibration plots for the internal validation of nomogram, the x-axis represents the nomogram predicted OS and the y-axis represents the actual OS of patients with LUAD.

A prognostic risk score model based on hub survival genes was created. According to the KM method, a higher risk score was linked to a significantly lower OS in LUAD (Figure 9C). The AUC plot showed that this prognostic signature had relatively high ability to predict the OS of LUAD patients (AUC > 0.7 for 1-year, 2-year and 3-year prediction, Figure 9D). These conclusions were validated in the TCGA-LUAD validation group (Figure 10A,B), total TCGA-LUAD group (Figure 10C,D), and an external GEO-LUAD group (Figure 10E,F). Notably, when we conducted the predictive analysis, there were only 5 genes (B3GALT2, CCR6, ABCC12, FOSL1, and FAM83A) in the GEO database. The detailed prognostic information of those genes was listed in a forest plot (Figure 10G). We further validated the prognostic results of these five genes by Kaplan–Meier plotter (KMPLOT) website [24], which showed consistent results as in the GEO database that the higher expression of B3GALT2 and CCR6 were associated with better OS of LUAD and the higher expression of FOSL1 and FAM83A were associated with worse OS of LUAD (Figure 10H). Moreover, a nomogram was constructed to predict 1-, 2-, and 3-year OS probability among individuals with LUAD, which comprised clinical features of age, gender, tumor stage, KRAS status and prognostic risk score model (Figure 11A). The calibration plots showed this nomogram had well prediction to the OS of LUAD patients (Figure 11B).

## 4. Discussion

The mitophagy was found to be association with a series of process that related to cancer, such as innate immunity [10], metabolism [10] and improvement of immunotherapy [25]. However, most of the research focused on just one or two mitophagy-related genes. A comprehensive analysis of multiple mitophagy genes in a cancer was required. To our best knowledge, there was no previous study on the association between mitophagy and LUAD. In our study, we found that the expression of 24 out of 27 mitophagy genes were significantly different between tumor and normal samples. The LUAD patients with distinct expression pattern of mitophagy genes showed significant prognostic difference. Further prognostic analyses identified key survival related DEGs of mitophagy groups. The risk score model based on key survival genes showed well prediction ability for the OS of LUAD.

We found that the CNV gains were associated with higher expression of mitophagy genes in 23 of 27 mitophagy genes. Moreover, we observed that the higher risk mitophagy groups were associated with significant higher total CNVs, which was reported to be a prognostic factor for cancer [26].

We discovered that there was a higher TMB in the high risk mitophagy group. Based on data from over 10,000 patients who did not receive therapy with immunological check point inhibitors (ICIs), higher TMB was found to be associated with worse survival in many malignancies [27]. Higher TMB was linked to more oncogenic drivers or mutations that could lead to therapeutic resistance [28], and higher intratumor genetic heterogeneity may have the potential to accelerate tumor growth in response to selective pressure [29]. However, it was found that higher TMB was associated with higher rates of treatment response and longer survival among patients who received the treatment of ICIs, which might attribute to higher numbers of potentially immunogenic neoantigens that may facilitate anti-tumor immune responses [30]. Furthermore, we found that the CD274 (Encode PD-L1 protein), PDCD1 (Encode PD-L1 protein) and LAG3 (a foremost immune therapy targets next to PD-1/PD-L1) were higher expressed in high risk mitophagy group. Previous study found impaired PINK1 and PRKN expression could promote the degradation of SLC25A37 and SLC25A28 and increase the mitochondrial iron accumulation, which lead to AIM2-mediated HMGB1 release that further induced expression of CD274/PD-L1 in tumor cells [31]. This result was consistent with ours, as the PINK1 and PRKN expression were significantly lower in the high risk mitophagy group. Further association between mitophagy and immune check points-related genes are required. In addition, we found that the CD4+ T cells were lower in the high risk mitophagy group. Previous study showed that PD-L1 inhibitor could induce expansion of tumor-infiltrating CD4+ and CD8+ T-cell subsets [32]. The above results indicated that the patients with certain mitophagy gene expression pattern as high risk mitophagy group might benefit from ICIs.

Cancer development, progression, and metastasis are significantly influenced by cancer stem cells [33]. Cancer cell stemness is also a significant contributor to therapeutic resistance [34]. We discovered that the high risk mitophagy group had a higher stemness score, which may be one of the causes of this group’s poorer prognosis.

PI3K/Akt/mTOR pathway was found to regulate the proliferation, apoptosis, metastasis of lung cancer, and various drugs that inhibit the PI3K axis are currently being tested in a series of clinical trials [35]. The PI3K/AKT/mTOR inhibitors wortmannin, LY-294002, and sirolimus could be possible treatments for the high risk mitophagy group, according to the CMap analysis of our study. By inducing the deacetylation of histone proteins, histone deacetylases (HDACs) play a crucial part in the regulation of transcription [36]. HDAC activation was widely proved to be associated with the resistance of chemotherapy, targeted therapy, and ICI therapy in cancers [37]. HDAC inhibitors (HDACi) has shown anti-proliferative activity in NSCLC cell lines [38]. A number of clinical studies showed that combined vorinostat and other treatments for LUAD might improve the effectiveness or reverse therapy resistance [37]. Our CMap analysis showed that HDACi trichostatin A and vorinostat had a potential therapeutic effect on LUAD patients in high risk mitophagy group. Furthermore, we also found that the high risk mitophagy group patients were more sensitive to cisplatin and gemcitabine, which were frequently recommended by the 2022-NSCLC-NCCN guideline as general chemotherapy agents for LUAD patients. Our drug sensitivity analysis provided new hints to assign the association between the systemic therapy of LUAD and mitophagy.

We further explored the DEGs and made functional enrichment analysis between two mitophagy groups. The KEGG analysis and GSEA analysis both revealed that cell cycle and oocyte meiosis were associated with high risk mitophagy group. The activation of TBK1 at the mitochondria during mitophagy by PINK1 and PRKN was found to cause a delay in mitosis because TBK1 was sequestered from its physiological function at centrosomes, however, if PINK1 and PRKN were not present, the cell cycle continued to progress [39]. Meiotic factors were found to promote tumor maintenance and therapeutic resistance by driving rapid tumor evolution [40]. Activation of the PRKN-mediated mitophagy pathway, lead to defects in meiosis and the accumulation of damaged mitochondria in oocytes [41]. The above studies were consistent with our results that PINK1 and PRKN were significantly lower in the high risk mitophagy group.

We further identified the mitophagy-related hub survival genes in LUAD. The constructed risk score model showed well predictive ability for LUAD OS and was validated by GEO database. To be noted, the genes in GEO database were not equivalent to the TCGA database. There were only five genes in the GEO database, which were B3GALB2, CCR6, ABCC12, FOSL1 and FAM83A. Among them, the B3GALB2, CCR6, FOSL1 and FAM83A were observed to have consistent prognostic results in three different sources of databases (TCGA, GEO and KMPLOT). CCR6 is the unique receptor for the chemokine CCL20 [42]. The effects of CCR6 on tumor progression and prognosis were controversial. Reports showed that the CCL20-CCR6 axis was associated with several cancers. It promoted cancer progression by enhancing migration and proliferation of cancer cells and remodeling the tumor microenvironment [43]. On the other hand, previous study showed a consistent pattern of CCR6 down-regulation in the cell lines and tissues of metastatic head and neck squamous cell carcinoma [44]. Additionally, transfection of Lewis lung carcinoma cells with CCR6 caused local production of CCL20 in the lung and reduced the metastatic possibility in mice [45]. In addition, a study with 84 patients of LUAD showed that higher expression of CCR6 in tumor was an independent predictor of a better prognosis in LUAD [46]. FOSL1 is a member of the FOS family, playing an important role in cancer cell progression in several cell types [47]. KRAS oncogene-induced FOSL1 activation was found to promote increased expression of AREG, cyclin D1, BCL2, and BCLXL, which were required for cell proliferation and cell survival [48]. Furthermore, FOSL1 genetic inhibition was found to be detrimental to KRAS-driven cancers such as LUAD and pancreatic adenocarcinoma [49]. Elevated FAM83A expression was observed to predict a poorer clinical outcome in LUAD [50]. It was found that lncRNA FAM83A-AS1 increased FAM83A expression by enhancing FAM83A pre-mRNA stability and promoted the tumorigenesis of LUAD [51]. FAM83A can inhibit GSK3β activity and increase the level of active unphosphorylated β-catenin; active β-catenin then transports into the nucleus and activates the Wnt signaling pathway in lung cancer cells [52]. B3GALT2 was found to be upregulated by tumor suppressor TGF-β [53]. Rare research was conducted between B3GALT2 and cancer. In summary, our study proved new insights between these genes and mitophagy and the prognosis of LUAD.

Our study had certain limitations. First, the data source from our study were all retrospectively designed, and with incomplete information such as the detail of therapy. Second, there was a lack of validation to the hub survival genes in in vitro or in vivo studies to explore the underlying mechanisms.

## 5. Conclusions

We first performed a comprehensive analysis of the landscape of 27 mitophagy genes by evaluating the RNA expression, CNVs, mutation status and clinicopathological features in LUAD. The expression pattern of mitophagy genes was significantly associated with the prognosis of LUAD and might influence the strategy of LUAD therapy. A mitophagy-related RNA signature was constructed and validated, which showed well predict ability for the prognosis of LUAD patients. Future studies are required to confirm the association between the hub survival genes and mitophagy and LUAD prognosis.

## Figures and Tables

**Table 1:** The potential drugs that targeted to high risk mitophagy group.

CMap Analysis			oncoPredict Analysis			
Drug	*p* value	FDR	Drug	High	Low	*p* value
trichostatin A_MCF7	5.76 × 10−42	2.07 × 10−38	Cisplatin_1005	8.39	40.87	4.28 × 10−15
trichostatin A_PC3	4.31 × 10−31	7.72 × 10−28	VE821_2111	26.94	64.51	1.01 × 10−14
LY-294002_MCF7	6.34 × 10−12	7.58 × 10−09	Doramapimod_1042	122.65	78.78	1.45 × 10−14
sirolimus_MCF7	1.11 × 10−09	9.97 × 10−07	Savolitinib_1936	5.35	15.39	1.89 × 10−14
tanespimycin_HL60	1.09 × 10−08	7.77 × 10−06	AZD7762_1022	0.40	1.27	2.17 × 10−14
fulvestrant_MCF7	1.30 × 10−08	7.77 × 10−06	Gemcitabine_1190	0.22	0.89	4.53 × 10−14
tanespimycin_MCF7	1.38 × 10−07	7.09 × 10−05	BMS.754807_2171	3.55	1.09	4.82 × 10−14
LY-294002_PC3	2.31 × 10−07	9.35 × 10−05	Ribociclib_1632	61.55	41.47	3.18 × 10−13
trichostatin A_HL60	2.35 × 10−07	9.35 × 10−05	MK.1775_1179	0.64	1.82	6.37 × 10−13
sirolimus_PC3	5.95 × 10−07	2.13 × 10−04	AZD6738_1917	3.03	9.79	6.59 × 10−13

## Data Availability

We downloaded the relative data from the Xena database (The latest accessed date was 1 September 2022. https://xenabrowser.net/datapages/) and GEO database (The latest accessed date was 1 September 2022. https://www.ncbi.nlm.nih.gov/geo/query/acc.cgi?acc=GSE72094). The KM plots of B3GALT2, CCR6, ABCC12, FOSL1, and FAM83A were also obtained from Kaplan–Meier plotter website (The latest accessed date was 1 September 2022. http://kmplot.com/analysis/).

## Supplementary Materials

The following supporting information can be downloaded at: https://www.mdpi.com/article/10.3390/cancers15010057/s1. Figure S1. The landscape of mutation of mitophagy genes in LUAD. The mutation frequency of mitophagy gene in LUAD patients from TCGA. Each column represented individual patients. The upper barplot showed the number of mutations per Mb, and the mutation rate of each gene was listed on the left. Table S1. The incidence rate of CNVs of mitophagy genes in TCGA-LUAD cohort. Table S2. The incidence rate of mutations of mitophagy genes in TCGA-LUAD cohort. Table S3. The DEGs identified between mitophagy groups in TCGA-LUAD training cohort. Table S4. Survival-related DEGs in TCGA-LUAD training cohort. Table S5. The coefficients of each survival-related DEG by LASSO analysis.
