# Supplementary material for: A Comprehensive Analysis of the Effects of Key Mitophagy Genes on the Progression and Prognosis of Lung Adenocarcinoma
Source: Cancers (Basel). 2022 Dec 22;15(1):57. doi: 10.3390/cancers15010057 (PMC9817891; doi:10.3390/cancers15010057)
Supplement: Supplementary file 1 [file cancers-15-00057-s001.zip › cancers-2038139-supplementary.pdf]

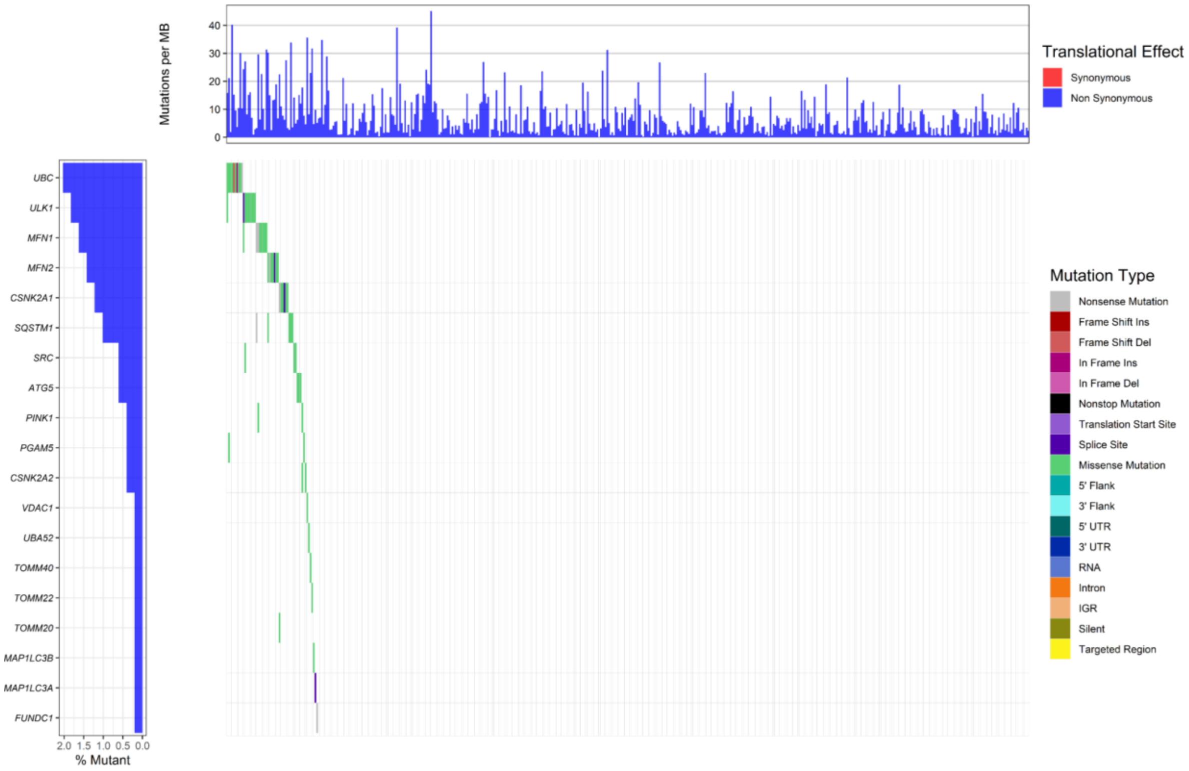

Figure S1. The landscape of mutation of mitophagy genes in LUAD. The mutation frequency of mitophagy gene in LUAD patients from TCGA. Each column represented individual patients. The upper barplot showed the number of mutations per Mb, and the mutation rate of each gene was listed on the left.

Gene	Loss (%)	Gain (%)
ATG12	5.84	1.36
ATG5	4.67	0.78
CSNK2A1	6.23	1.75
CSNK2A2	2.33	3.31
CSNK2B	0.97	6.23
FUNDC1	1.17	3.7
MAP1LC3A	2.92	5.25
MAP1LC3B	6.61	0.97
MFN1	1.95	7.39
MFN2	5.45	5.25
MTERF3	2.33	10.7
PGAM5	5.64	3.7
PINK1	6.03	3.89
PRKN	5.84	1.56
RPS27A	1.36	3.31
SQSTM1	3.11	7
SRC	3.11	4.28
TOMM20	2.14	5.84
TOMM22	1.75	4.09
TOMM40	4.28	7.39
TOMM5	3.89	4.67
TOMM7	0.78	6.42
UBA52	1.95	2.92
UBB	4.28	1.95
UBC	5.06	3.31
ULK1	5.84	4.28
VDAC1	6.61	0.19

Table S1. The incidence rate of CNVs of mitophagy genes in TCGA-LUAD cohort.

Gene	Frequency
UBC	2.03%
ULK1	1.83%
MFN1	1.63%
MFN2	1.42%
CSNK2A1	1.22%
SQSTM1	1.02%
ATG5	0.61%
SRC	0.61%
CSNK2A2	0.41%
PGAM5	0.41%
PINK1	0.41%
FUNDC1	0.20%
MAP1LC3A	0.20%
MAP1LC3B	0.20%
TOMM20	0.20%
TOMM22	0.20%
TOMM40	0.20%
UBA52	0.20%
VDAC1	0.20%
ATG12	0.20%
CSNK2B	0.00%
MTERF3	0.00%
PRKN	0.00%
RPS27A	0.00%
TOMM5	0.00%
TOMM7	0.00%
UBB	0.00%

Table S2. The incidence rate of mutations of mitophagy genes in TCGA-LUAD cohort.

Gene	logFC	t	adj.P.Val
BIRC5	1.86	15.08	9.84 × 10−35
UBE2C	2.05	14.98	1.16 × 10−34
NUF2	1.81	14.57	3.26 × 10−33
CDCA5	1.62	14.51	3.94 × 10−33
AURKB	1.74	14.49	3.94 × 10−33
TROAP	1.80	14.45	3.96 × 10−33
CENPA	1.90	14.45	3.96 × 10−33
MELK	1.86	14.39	6.09 × 10−33
DBF4	1.05	14.36	6.82 × 10−33
CDC20	1.73	14.21	2.26 × 10−32
TTK	1.76	14.19	2.61 × 10−32
EME1	1.49	13.99	1.33 × 10−31
CDKN3	1.72	13.95	1.78 × 10−31
DSCC1	1.41	13.91	2.25 × 10−31
CCNB2	1.51	13.85	3.68 × 10−31
AUNIP	1.59	13.78	6.54 × 10−31
CDK1	1.52	13.73	9.18 × 10−31
KPNA2	1.19	13.72	9.32 × 10−31
SPC24	1.42	13.71	9.92 × 10−31
RFC4	1.13	13.68	1.29 × 10−30
MCM10	1.86	13.64	1.67 × 10−30
HJURP	1.74	13.55	3.12 × 10−30
AURKA	1.51	13.54	3.12 × 10−30
SKA3	1.59	13.56	3.11 × 10−30
MTBP	1.12	13.55	3.12 × 10−30
PRKN	-1.57	-13.56	3.09 × 10−30
CDC45	1.60	13.44	7.16 × 10−30
MTFR2	1.48	13.46	6.45 × 10−30
CDCA8	1.41	13.36	1.42 × 10−29
SKA1	1.73	13.36	1.41 × 10−29
CENPW	1.44	13.34	1.59 × 10−29
TRAIP	1.02	13.32	1.83 × 10−29
ORC6	1.37	13.30	2.17 × 10−29
CEP55	1.57	13.19	5.15 × 10−29
CCNB1	1.38	13.18	5.65 × 10−29
TPX2	1.70	13.16	6.52 × 10−29

NDC80	1.62	13.15	6.52 × 10−29
TRIP13	1.60	13.14	6.95 × 10−29
PIMREG	1.71	13.13	7.71 × 10−29
KIF2C	1.60	13.10	9.56 × 10−29
CDCA3	1.47	13.08	1.15 × 10−28
CCNA2	1.47	13.02	1.76 × 10−28
KIF18B	1.71	12.99	2.30 × 10−28
SGO1	1.57	12.98	2.49 × 10−28
ARHGEF39	1.15	12.94	3.52 × 10−28
BUB1	1.49	12.91	4.29 × 10−28
KIF23	1.49	12.89	4.94 × 10−28
CHEK1	1.25	12.84	7.87 × 10−28
CDC6	1.52	12.83	8.04 × 10−28
RAD54B	1.09	12.79	1.17 × 10−27
TNS1	-1.18	-12.76	1.39 × 10−27
PLK4	1.25	12.76	1.42 × 10−27
NCAPG	1.62	12.75	1.46 × 10−27
SCN7A	-2.02	-12.75	1.45 × 10−27
SPAG5	1.35	12.73	1.70 × 10−27
SPC25	1.41	12.69	2.28 × 10−27
KIF15	1.44	12.68	2.48 × 10−27
PRR11	1.49	12.67	2.69 × 10−27
GINS1	1.34	12.60	4.29 × 10−27
NCAPH	1.52	12.60	4.29 × 10−27
CENPM	1.20	12.61	4.29 × 10−27
UBE2T	1.35	12.60	4.45 × 10−27
DEPDC1	1.76	12.57	5.56 × 10−27
POLE2	1.21	12.56	5.92 × 10−27
CKAP2L	1.57	12.50	9.79 × 10−27
XRCC2	1.41	12.50	1.00 × 10−26
ASF1B	1.24	12.48	1.16 × 10−26
DEPDC1B	1.61	12.48	1.12 × 10−26
KIF4A	1.61	12.46	1.26 × 10−26
MYBL2	1.86	12.46	1.29 × 10−26
DLGAP5	1.64	12.46	1.29 × 10−26
RAI2	-1.16	-12.43	1.58 × 10−26
C1QTNF7	-1.97	-12.46	1.30 × 10−26
TK1	1.33	12.39	2.19 × 10−26
NEK2	1.59	12.37	2.60 × 10−26
RAD51AP1	1.35	12.34	3.27 × 10−26
PSRC1	1.10	12.34	3.27 × 10−26
POC1A	1.00	12.33	3.39 × 10−26
RAD51	1.32	12.33	3.51 × 10−26
C5orf34	1.17	12.31	4.16 × 10−26
SAPCD2	1.43	12.26	6.00 × 10−26
CENPK	1.26	12.22	8.56 × 10−26
GTSE1	1.41	12.19	1.08 × 10−25
PARPBP	1.28	12.18	1.17 × 10−25
RAD54L	1.49	12.15	1.39 × 10−25
CDC25A	1.37	12.11	1.93 × 10−25
FAM72A	1.45	12.12	1.86 × 10−25
DPYSL2	-1.04	-12.05	3.36 × 10−25
UBE2S	1.23	12.04	3.62 × 10−25
ZWINT	1.19	12.03	3.63 × 10−25
FAM72D	1.73	12.06	2.94 × 10−25
CENPE	1.43	11.95	6.92 × 10−25
CDC25C	1.49	11.95	7.03 × 10−25
FAM72B	1.65	11.97	5.83 × 10−25
SGO2	1.14	11.93	8.02 × 10−25
CKS2	1.22	11.88	1.22 × 10−24
EZH2	1.09	11.87	1.24 × 10−24
CDCA2	1.45	11.88	1.22 × 10−24
MAD2L1	1.35	11.85	1.45 × 10−24
PLK1	1.31	11.84	1.59 × 10−24
C17orf53	1.32	11.84	1.52 × 10−24
TEDC2	1.18	11.83	1.66 × 10−24
EXO1	1.56	11.81	2.02 × 10−24
KIFC1	1.39	11.80	2.13 × 10−24
PCLAF	1.32	11.80	2.14 × 10−24

ELN	-1.74	-11.76	2.79×10^{-24}
FANCB	1.23	11.79	2.26×10^{-24}
CIP2A	1.32	11.74	3.31×10^{-24}
CCNE1	1.48	11.71	4.20×10^{-24}
ANLN	1.60	11.70	4.39×10^{-24}
KIF20A	1.32	11.70	4.49×10^{-24}
POLQ	1.52	11.70	4.39×10^{-24}
STIL	1.08	11.69	4.77×10^{-24}
KIF11	1.23	11.65	6.75×10^{-24}
ORC1	1.40	11.59	1.08×10^{-23}
BUB1B	1.40	11.58	1.13×10^{-23}
ADAMTS8	-2.09	-11.60	1.02×10^{-23}
KIF14	1.56	11.57	1.26×10^{-23}
NUSAP1	1.19	11.55	1.38×10^{-23}
HASPIN	1.32	11.54	1.52×10^{-23}
OIP5	1.39	11.54	1.52×10^{-23}
TOP2A	1.45	11.52	1.84×10^{-23}
PKMYT1	1.35	11.51	1.94×10^{-23}
PRC1	1.19	11.48	2.42×10^{-23}
RACGAP1	1.05	11.46	2.87×10^{-23}
PIF1	1.38	11.45	3.12×10^{-23}
ITGA8	-1.38	-11.44	3.42×10^{-23}
ASPM	1.52	11.43	3.54×10^{-23}
RRM2	1.42	11.43	3.57×10^{-23}
ABCA8	-1.86	-11.39	4.68×10^{-23}
CENPI	1.39	11.39	4.94×10^{-23}
MCM4	1.08	11.37	5.68×10^{-23}
CDT1	1.28	11.33	7.55×10^{-23}
KIF18A	1.14	11.31	8.82×10^{-23}
ROBO2	-2.03	-11.30	9.47×10^{-23}
TICRR	1.41	11.30	9.94×10^{-23}
AC099850.3	1.52	11.28	1.17×10^{-22}
SCN4B	-1.50	-11.24	1.49×10^{-22}
MND1	1.20	11.19	2.26×10^{-22}
ESPL1	1.46	11.18	2.49×10^{-22}
INMT	-1.82	-11.16	2.75×10^{-22}
ADAMTSL2	-1.14	-11.16	2.91×10^{-22}
RECQL4	1.22	11.14	3.33×10^{-22}
HMMR	1.36	11.10	4.60×10^{-22}
FOXM1	1.45	11.06	6.36×10^{-22}
AC012073.1	1.03	11.08	5.41×10^{-22}
MFAP4	-1.67	-11.03	8.03×10^{-22}
CCNE2	1.13	10.96	1.40×10^{-21}
ECE2	1.00	10.94	1.54×10^{-21}
GINS4	1.23	10.94	1.54×10^{-21}
MGP	-1.36	-10.91	2.06×10^{-21}
ATP1A2	-1.93	-10.92	1.90×10^{-21}
EIF4EBP1	1.12	10.86	2.91×10^{-21}
HELLS	1.07	10.86	2.98×10^{-21}
BRCA1	1.11	10.85	3.19×10^{-21}
MYOCD	-1.90	-10.87	2.75×10^{-21}
MAP6D1	1.12	10.85	3.22×10^{-21}
DDIAS	1.29	10.82	3.98×10^{-21}
MYH11	-1.65	-10.81	4.13×10^{-21}
BRIP1	1.21	10.77	5.74×10^{-21}
NFIX	-1.23	-10.75	6.76×10^{-21}
ATAD2	1.02	10.73	7.70×10^{-21}
TYMS	1.03	10.71	8.98×10^{-21}
AOC3	-1.24	-10.71	9.19×10^{-21}
KNL1	1.22	10.66	1.33×10^{-20}
DNA2	1.03	10.65	1.43×10^{-20}
PBK	1.57	10.64	1.58×10^{-20}
HMCN1	-1.29	-10.58	2.42×10^{-20}
NR3C2	-1.42	-10.56	2.78×10^{-20}
LAMA2	-1.10	-10.56	2.80×10^{-20}
CCDC150	1.23	10.52	3.68×10^{-20}
WDHD1	1.00	10.49	4.58×10^{-20}
SHCBP1	1.15	10.49	4.60×10^{-20}
MAMDC2	-1.73	-10.49	4.58×10^{-20}

SGCA	-1.46	-10.49	4.60×10^{-20}
ARHGAP11A	1.13	10.45	6.43×10^{-20}
ERCC6L	1.23	10.45	6.46×10^{-20}
A2M	-1.20	-10.48	5.20×10^{-20}
ADH1B	-2.88	-10.40	9.20×10^{-20}
SLIT3	-1.52	-10.38	1.07×10^{-19}
NTRK3	-1.50	-10.39	9.71×10^{-20}
ITGA9	-1.21	-10.37	1.14×10^{-19}
SNED1	-1.01	-10.37	1.16×10^{-19}
E2F1	1.11	10.31	1.86×10^{-19}
C7	-2.15	-10.29	2.05×10^{-19}
TBX4	-1.45	-10.26	2.59×10^{-19}
UHRF1	1.26	10.24	2.94×10^{-19}
CENPU	1.11	10.20	4.09×10^{-19}
DIAPH3	1.22	10.19	4.25×10^{-19}
GFRA1	-1.93	-10.15	6.00×10^{-19}
PTTG1	1.14	10.11	8.29×10^{-19}
ESCO2	1.20	10.11	8.29×10^{-19}
ITIH5	-1.53	-10.10	8.39×10^{-19}
SPARCL1	-1.05	-10.10	8.82×10^{-19}
PSAT1	1.41	10.07	1.03×10^{-18}
TBX5-AS1	-1.10	-10.06	1.12×10^{-18}
FAM72C	1.73	10.05	1.17×10^{-18}
SPATA18	-1.77	-9.99	1.84×10^{-18}
WDR62	1.19	9.98	1.96×10^{-18}
CTSV	1.70	9.96	2.35×10^{-18}
GINS2	1.16	9.96	2.41×10^{-18}
GRIA1	-2.35	-9.97	2.16×10^{-18}
TMEM132E	-1.64	-9.93	3.00×10^{-18}
AC120049.1	-1.02	-9.92	3.21×10^{-18}
FENDRR	-1.62	-9.86	4.82×10^{-18}
HPSE2	-1.96	-9.87	4.32×10^{-18}
PODN	-1.14	-9.85	5.19×10^{-18}
LTBP2	-1.13	-9.86	4.82×10^{-18}
CFAP221	-1.85	-9.79	8.11×10^{-18}
CGNL1	-1.05	-9.74	1.18×10^{-17}
SHROOM4	-1.09	-9.70	1.59×10^{-17}
ACRV1	1.41	9.69	1.62×10^{-17}
DTL	1.07	9.65	2.29×10^{-17}
PGR	-1.21	-9.65	2.21×10^{-17}
E2F2	1.08	9.59	3.45×10^{-17}
CYP4B1	-2.62	-9.58	3.84×10^{-17}
NEIL3	1.60	9.52	5.58×10^{-17}
SLC5A9	-1.57	-9.53	5.28×10^{-17}
PKNOX2	-1.22	-9.51	6.18×10^{-17}
VWF	-1.13	-9.51	5.98×10^{-17}
IQGAP3	1.15	9.49	6.98×10^{-17}
FGF14	-1.19	-9.49	7.09×10^{-17}
RPL39L	1.30	9.48	7.44×10^{-17}
RSPO2	-2.29	-9.48	7.23×10^{-17}
HSPB7	-1.11	-9.41	1.20×10^{-16}
SULT1C4	-1.08	-9.42	1.16×10^{-16}
LPAL2	-1.45	-9.42	1.15×10^{-16}
MAOB	-1.26	-9.40	1.30×10^{-16}
POLR3G	1.01	9.38	1.53×10^{-16}
PHACTR1	-1.05	-9.37	1.55×10^{-16}
ASPA	-1.24	-9.39	1.44×10^{-16}
CACNA2D2	-2.20	-9.37	1.55×10^{-16}
ATP5PBP5	-1.28	-9.38	1.47×10^{-16}
UBE2SP1	1.38	9.36	1.65×10^{-16}
FAM83D	1.24	9.34	2.01×10^{-16}
RCAN2	-1.14	-9.33	2.06×10^{-16}
TCF21	-1.47	-9.33	2.13×10^{-16}
ADAMTSL3	-1.23	-9.33	2.15×10^{-16}
RAP1GAP	-1.58	-9.33	2.15×10^{-16}
SLC22A3	-1.91	-9.29	2.89×10^{-16}
FBXO43	1.13	9.29	2.72×10^{-16}
ZBTB16	-2.22	-9.28	3.03×10^{-16}
PRDM6	-1.03	-9.27	3.11×10^{-16}

CENPF	1.24	9.28	2.98 × 10−16
VEGFD	-2.28	-9.27	3.08 × 10−16
GPR19	1.22	9.28	3.04 × 10−16
F11	-2.70	-9.28	3.04 × 10−16
NDNF	-1.80	-9.22	4.39 × 10−16
KCND3	-1.30	-9.18	5.73 × 10−16
CORO2B	-1.07	-9.18	6.10 × 10−16
CYBRD1	-1.09	-9.20	5.33 × 10−16
C16orf89	-2.47	-9.19	5.58 × 10−16
LMOD1	-1.01	-9.17	6.34 × 10−16
AC236972.3	-2.49	-9.19	5.68 × 10−16
DLC1	-1.24	-9.13	8.18 × 10−16
SLC44A4	-1.40	-9.14	7.85 × 10−16
TYMSOS	1.22	9.10	9.99 × 10−16
DNMT3B	1.09	9.06	1.35 × 10−15
VWA2	-1.33	-9.06	1.35 × 10−15
LINC00337	1.66	9.08	1.22 × 10−15
LINC01936	-1.25	-9.05	1.40 × 10−15
LINC00261	-2.63	-9.04	1.52 × 10−15
ABI3BP	-1.28	-9.05	1.49 × 10−15
ART4	-1.49	-9.04	1.51 × 10−15
FHL1	-1.25	-9.02	1.75 × 10−15
LINC00634	1.62	9.04	1.60 × 10−15
KCNA5	-1.24	-9.01	1.96 × 10−15
HLF	-1.73	-8.98	2.32 × 10−15
C2orf40	-2.22	-8.99	2.24 × 10−15
PCDH20	-2.08	-8.98	2.30 × 10−15
AC023509.2	-1.63	-8.97	2.46 × 10−15
CLSPN	1.25	8.93	3.28 × 10−15
ADGRF5	-1.43	-8.94	3.08 × 10−15
PLA2G1B	-2.48	-8.90	3.88 × 10−15
LINC00940	-1.97	-8.88	4.58 × 10−15
FOXA2	-2.17	-8.87	4.80 × 10−15
MAP6	-1.13	-8.86	5.32 × 10−15
EDNRB	-1.13	-8.86	5.37 × 10−15
CX3CR1	-1.52	-8.85	5.50 × 10−15
TNXB	-1.44	-8.85	5.50 × 10−15
PRELP	-1.19	-8.86	5.18 × 10−15
MKI67	1.20	8.83	6.30 × 10−15
PLXNA2	-1.06	-8.83	6.45 × 10−15
TESMIN	1.53	8.80	8.00 × 10−15
BTG2	-1.02	-8.82	6.78 × 10−15
HPDL	1.63	8.78	8.86 × 10−15
FMO2	-1.26	-8.78	9.06 × 10−15
MEGF6	-1.09	-8.78	9.20 × 10−15
NEGR1	-1.30	-8.71	1.46 × 10−14
MS4A2	-1.56	-8.68	1.75 × 10−14
RHOBTB2	-1.16	-8.71	1.50 × 10−14
SELENBP1	-1.36	-8.71	1.46 × 10−14
KLHL33	-1.29	-8.69	1.63 × 10−14
FXYD1	-1.14	-8.65	2.16 × 10−14
EDA2R	-1.51	-8.63	2.44 × 10−14
CRTAC1	-2.00	-8.63	2.50 × 10−14
CAV3	-1.54	-8.62	2.61 × 10−14
CCL14	-1.58	-8.63	2.58 × 10−14
CHRDL1	-1.75	-8.62	2.68 × 10−14
SVEP1	-1.23	-8.61	2.90 × 10−14
CST5	-1.90	-8.61	2.76 × 10−14
FAM111B	1.09	8.59	3.14 × 10−14
SHH	-1.91	-8.58	3.44 × 10−14
LINC01266	-1.24	-8.59	3.20 × 10−14
SCN11A	-1.17	-8.59	3.32 × 10−14
DDN	1.54	8.56	3.85 × 10−14
RDM1	1.33	8.54	4.39 × 10−14
CYP4Z2P	-2.15	-8.55	4.13 × 10−14
SCTR	-2.39	-8.53	4.92 × 10−14
SLC8A3	-1.30	-8.54	4.57 × 10−14
PEBP4	-2.53	-8.51	5.28 × 10−14
BTNL9	-1.37	-8.50	5.60 × 10−14

CASQ2	-1.55	-8.51	5.31 × 10−14
E2F7	1.28	8.50	5.90 × 10−14
UNC45B	-1.18	-8.51	5.47 × 10−14
CLEC4F	-1.69	-8.50	5.69 × 10−14
FOLR1	-2.00	-8.51	5.50 × 10−14
FHL5	-1.21	-8.47	7.03 × 10−14
C8orf34	-1.51	-8.47	7.06 × 10−14
AC091948.1	-1.14	-8.48	6.63 × 10−14
E2F8	1.03	8.45	8.24 × 10−14
INSYN1	-1.42	-8.44	8.57 × 10−14
SELENOP	-1.09	-8.46	7.36 × 10−14
VWC2	-1.49	-8.46	7.69 × 10−14
FCER1A	-2.04	-8.42	1.01 × 10−13
MACROD2	-1.71	-8.41	1.02 × 10−13
ADAMTS7P3	-1.64	-8.42	9.87 × 10−14
SFTPD	-2.29	-8.41	1.02 × 10−13
AC073585.1	1.26	8.37	1.35 × 10−13
AR	-1.34	-8.37	1.40 × 10−13
ERBB4	-1.77	-8.36	1.42 × 10−13
MAPK10	-1.20	-8.35	1.58 × 10−13
NPM1P9	1.01	8.36	1.45 × 10−13
GATA5	-1.37	-8.34	1.68 × 10−13
TMEM132D	-2.70	-8.33	1.71 × 10−13
AC022164.1	-1.28	-8.32	1.90 × 10−13
CTSG	-1.82	-8.29	2.22 × 10−13
GPIHBP1	-1.43	-8.28	2.44 × 10−13
LINC01354	-1.30	-8.26	2.75 × 10−13
SFTPB	-2.36	-8.35	1.54 × 10−13
CPA3	-1.55	-8.24	3.12 × 10−13
PARM1	-1.27	-8.25	2.85 × 10−13
CD300LG	-1.97	-8.23	3.40 × 10−13
PLAC1	1.96	8.21	3.71 × 10−13
ADGRF5P2	-1.49	-8.22	3.59 × 10−13
ROS1	-1.89	-8.22	3.59 × 10−13
FPGT-TNNI3K	-1.23	-8.19	4.18 × 10−13
CASR	-2.42	-8.19	4.15 × 10−13
AL138789.1	1.48	8.18	4.40 × 10−13
GDF10	-1.61	-8.17	4.82 × 10−13
PRDM16	-1.68	-8.17	4.79 × 10−13
C1orf116	-1.48	-8.19	4.20 × 10−13
LINC00968	-1.31	-8.15	5.60 × 10−13
FAT4	-1.02	-8.14	5.98 × 10−13
ABCA3	-1.52	-8.13	6.11 × 10−13
GALNT17	-1.45	-8.08	8.49 × 10−13
LARGE1	-1.05	-8.09	8.34 × 10−13
GGTLC1	-2.43	-8.06	9.76 × 10−13
LINC02519	-1.28	-8.06	9.53 × 10−13
TMPRSS2	-1.31	-8.09	8.26 × 10−13
CPAMD8	-1.57	-8.06	9.63 × 10−13
ANGPT1	-1.16	-8.05	1.04 × 10−12
UBE2SP2	1.33	8.06	9.88 × 10−13
ADH1A	-1.38	-8.03	1.15 × 10−12
AFF3	-1.43	-8.02	1.24 × 10−12
ZNF366	-1.00	-8.02	1.25 × 10−12
TSACC	1.05	8.02	1.25 × 10−12
KBTBD11	-1.09	-8.02	1.25 × 10−12
C8orf34-AS1	-2.23	-8.02	1.29 × 10−12
CYP2B7P	-2.06	-8.04	1.12 × 10−12
SLC26A9	-2.11	-8.02	1.25 × 10−12
GJB1	-2.60	-8.00	1.41 × 10−12
LINC00982	-1.80	-8.00	1.42 × 10−12
NAPSA	-2.14	-8.07	9.30 × 10−13
FGF10	-1.69	-7.99	1.48 × 10−12
DUOX1	-1.47	-7.99	1.52 × 10−12
ATP13A4	-1.91	-7.98	1.67 × 10−12
SSTR1	-1.73	-7.96	1.79 × 10−12
OGN	-1.73	-7.96	1.85 × 10−12
C4BPA	-2.12	-7.96	1.81 × 10−12
ZNF887P	1.08	7.94	2.06 × 10−12

HPGDS	-1.35	-7.90	2.77 × 10−12
ACADL	-1.78	-7.89	2.88 × 10−12
KCNQ1	-1.17	-7.90	2.68 × 10−12
AP001189.1	-1.09	-7.89	2.88 × 10−12
AL138900.2	-1.83	-7.88	3.10 × 10−12
SUSD2	-1.82	-7.87	3.22 × 10−12
ALKAL2	-1.29	-7.84	3.95 × 10−12
ACTN2	-1.15	-7.84	4.04 × 10−12
CLDN18	-3.00	-7.82	4.40 × 10−12
SMCO3	-1.00	-7.80	5.11 × 10−12
TNR	-1.43	-7.79	5.33 × 10−12
LRRK2	-1.67	-7.81	4.90 × 10−12
MYOM2	-1.11	-7.78	5.71 × 10−12
ADRA1A	-1.62	-7.78	5.54 × 10−12
PTPRU	-1.06	-7.80	5.09 × 10−12
AC096531.2	-1.84	-7.77	5.86 × 10−12
AC005884.1	-1.24	-7.77	6.11 × 10−12
SCGB3A2	-2.93	-7.78	5.53 × 10−12
ADGRD1	-1.37	-7.77	5.94 × 10−12
AC037441.1	-1.60	-7.77	6.14 × 10−12
COLEC12	-1.09	-7.77	6.07 × 10−12
FAM107A	-1.26	-7.75	6.94 × 10−12
SCN2B	-1.32	-7.73	7.68 × 10−12
PTCHD4	-1.57	-7.72	8.21 × 10−12
B3GALT2	-1.33	-7.71	8.47 × 10−12
PGM5	-1.12	-7.72	8.30 × 10−12
CNTN6	-1.82	-7.68	1.04 × 10−11
PRG4	-1.79	-7.68	1.06 × 10−11
SPTLC3	-1.25	-7.69	1.00 × 10−11
CTXND1	-1.51	-7.67	1.09 × 10−11
ZNF695	1.85	7.67	1.12 × 10−11
LINC02038	-1.91	-7.66	1.16 × 10−11
CD1E	-1.69	-7.66	1.19 × 10−11
SEC14L6	-1.47	-7.65	1.22 × 10−11
C9orf152	-1.43	-7.66	1.17 × 10−11
VSIG2	-1.87	-7.66	1.21 × 10−11
AC093797.1	-1.38	-7.64	1.30 × 10−11
PACRG	-1.62	-7.64	1.33 × 10−11
RSPO1	-1.36	-7.64	1.32 × 10−11
MEOX2	-1.04	-7.62	1.49 × 10−11
HMCN2	-1.22	-7.61	1.59 × 10−11
COL14A1	-1.17	-7.63	1.39 × 10−11
TMEM252	-1.42	-7.61	1.62 × 10−11
AC090092.1	-1.22	-7.61	1.65 × 10−11
LHFPL3-AS2	-2.19	-7.58	1.89 × 10−11
DUOXA1	-1.50	-7.58	1.88 × 10−11
TMEM132C	-1.84	-7.57	2.01 × 10−11
PLCXD3	-1.66	-7.56	2.17 × 10−11
TMEM163	-1.33	-7.58	1.95 × 10−11
AC135012.3	-1.29	-7.56	2.16 × 10−11
DIO3OS	-1.62	-7.55	2.25 × 10−11
AL157996.1	-1.28	-7.54	2.40 × 10−11
C4B	-1.17	-7.54	2.44 × 10−11
RPL13AP17	-2.15	-7.53	2.58 × 10−11
SCNN1B	-1.42	-7.54	2.43 × 10−11
SORBS2	-1.21	-7.53	2.63 × 10−11
RCOR2	1.33	7.48	3.59 × 10−11
GPRC5C	-1.07	-7.50	3.06 × 10−11
DBF4P1	1.11	7.47	3.75 × 10−11
NALCN	-1.02	-7.45	4.27 × 10−11
LINC01775	1.17	7.44	4.64 × 10−11
LGI3	-2.43	-7.43	4.81 × 10−11
AL390961.3	1.07	7.42	4.93 × 10−11
AC112777.1	1.21	7.42	4.97 × 10−11
AC018647.1	-1.05	-7.42	5.01 × 10−11
WSCD2	-1.28	-7.42	5.16 × 10−11
LINC01352	-1.10	-7.42	5.16 × 10−11
GLI1	-1.07	-7.41	5.48 × 10−11
PGM5P4	-1.51	-7.40	5.75 × 10−11

AC005165.1	-1.64	-7.39	6.12×10^{-11}
FMO5	-1.31	-7.40	5.60×10^{-11}
AC013275.1	-1.92	-7.38	6.62×10^{-11}
HIST1H3B	1.64	7.38	6.48×10^{-11}
ISLR2	-1.09	-7.37	6.78×10^{-11}
AC005042.1	1.22	7.38	6.54×10^{-11}
SFTA1P	-1.81	-7.37	6.64×10^{-11}
AP003555.3	-1.26	-7.37	6.97×10^{-11}
COL6A6	-1.44	-7.36	7.05×10^{-11}
LINC01213	1.54	7.36	7.21×10^{-11}
AC009041.1	-1.26	-7.36	7.32×10^{-11}
CD207	-2.17	-7.35	7.46×10^{-11}
CAPN8	-1.60	-7.39	6.20×10^{-11}
CAVIN2	-1.02	-7.37	6.66×10^{-11}
PRSS12	-1.65	-7.34	8.16×10^{-11}
AL731557.1	-1.24	-7.33	8.74×10^{-11}
HIST2H2AC	1.13	7.32	8.97×10^{-11}
PNMA2	-1.20	-7.34	8.04×10^{-11}
AC008878.3	-1.27	-7.32	9.03×10^{-11}
RAC3	1.01	7.33	8.34×10^{-11}
THSD7B	-1.28	-7.32	9.21×10^{-11}
CD1C	-1.37	-7.32	9.16×10^{-11}
ANKFN1	-1.70	-7.30	1.01×10^{-10}
LSP1P2	-1.15	-7.30	1.02×10^{-10}
ADCY2	-1.39	-7.30	1.05×10^{-10}
CLEC3B	-1.14	-7.29	1.10×10^{-10}
AL355388.1	-1.63	-7.28	1.15×10^{-10}
CTSH	-1.09	-7.36	7.33×10^{-11}
SCARA3	-1.01	-7.29	1.10×10^{-10}
HSD17B6	-1.38	-7.28	1.15×10^{-10}
RASGRF1	-1.66	-7.27	1.19×10^{-10}
LRRC31	-2.07	-7.26	1.28×10^{-10}
AC021945.1	1.14	7.26	1.30×10^{-10}
CDCA7	1.09	7.26	1.25×10^{-10}
PENK	-1.81	-7.24	1.46×10^{-10}
DES	-1.44	-7.24	1.46×10^{-10}
AFF2	-1.31	-7.23	1.55×10^{-10}
TPPP	-1.17	-7.24	1.41×10^{-10}
SHE	-1.26	-7.23	1.50×10^{-10}
LINC01942	-1.50	-7.21	1.70×10^{-10}
VIPR1	-1.08	-7.22	1.65×10^{-10}
FOS	-1.08	-7.27	1.19×10^{-10}
PTPN13	-1.15	-7.23	1.49×10^{-10}
FAM180A	-1.07	-7.19	1.90×10^{-10}
MMRN1	-1.13	-7.20	1.81×10^{-10}
AC104035.1	-1.27	-7.19	1.90×10^{-10}
TPSAB1	-1.28	-7.20	1.80×10^{-10}
HHIP	-2.12	-7.19	1.90×10^{-10}
CCR6	-1.24	-7.19	1.91×10^{-10}
PLA2G10	-1.69	-7.17	2.09×10^{-10}
EDA	-1.18	-7.17	2.09×10^{-10}
ITGB6	-1.07	-7.23	1.57×10^{-10}
HNF1B	-1.40	-7.19	1.94×10^{-10}
IRX1	-2.27	-7.16	2.23×10^{-10}
CTSE	-2.26	-7.20	1.78×10^{-10}
P3H2	-1.38	-7.17	2.09×10^{-10}
OTC	-1.38	-7.15	2.37×10^{-10}
AC137834.2	1.12	7.14	2.56×10^{-10}
KCNA4	-1.49	-7.14	2.51×10^{-10}
LINC02321	1.38	7.14	2.51×10^{-10}
HIST1H2AJ	1.45	7.14	2.58×10^{-10}
MAOA	-1.10	-7.18	2.00×10^{-10}
ITGBL1	-1.03	-7.15	2.39×10^{-10}
AC007787.2	-1.19	-7.13	2.75×10^{-10}
C1QL4	1.52	7.13	2.72×10^{-10}
OLFM1	-1.21	-7.13	2.66×10^{-10}
PTPRT	-2.17	-7.12	2.86×10^{-10}
ARTN	1.16	7.11	2.93×10^{-10}
LEFTY2	-1.53	-7.11	3.04×10^{-10}

GALNT16	-1.14	-7.11	3.06 × 10−10
WIF1	-2.47	-7.11	3.00 × 10−10
AL445423.1	-1.05	-7.11	3.02 × 10−10
MALL	-1.12	-7.12	2.78 × 10−10
MUSK	-1.28	-7.09	3.31 × 10−10
SHOX2	1.56	7.08	3.52 × 10−10
HABP2	-2.11	-7.09	3.44 × 10−10
CLU	-1.29	-7.15	2.39 × 10−10
MYOC	-1.46	-7.08	3.61 × 10−10
MUC1	-1.07	-7.18	2.02 × 10−10
DUSP27	-1.50	-7.07	3.79 × 10−10
CHIA	-2.25	-7.07	3.85 × 10−10
ERVFRD-1	-1.08	-7.06	3.93 × 10−10
PTCSC3	-1.78	-7.05	4.16 × 10−10
AL133466.1	-1.47	-7.05	4.17 × 10−10
VILL	-1.17	-7.07	3.79 × 10−10
FDPSP8	1.08	7.05	4.21 × 10−10
AP003385.4	-1.33	-7.05	4.24 × 10−10
MT1H	1.61	7.05	4.30 × 10−10
ATP13A4-AS1	-1.88	-7.05	4.23 × 10−10
DAAM2	-1.04	-7.07	3.85 × 10−10
ATP5MC1P4	1.01	7.04	4.41 × 10−10
HR	-1.20	-7.04	4.50 × 10−10
CEACAM8	-1.81	-7.04	4.43 × 10−10
ACKR1	-1.53	-7.05	4.26 × 10−10
AC009093.3	-1.54	-7.04	4.51 × 10−10
GUCA1A	1.49	7.03	4.66 × 10−10
CHIAP2	-2.32	-7.03	4.72 × 10−10
AC104260.2	-1.12	-7.03	4.71 × 10−10
AL162511.1	-1.75	-7.02	5.12 × 10−10
LINC01645	-1.21	-7.02	5.11 × 10−10
AC046195.1	-2.08	-7.02	5.12 × 10−10
MIR27A	-1.08	-7.01	5.32 × 10−10
CAPN3	-1.02	-7.01	5.35 × 10−10
RAB44	-1.05	-7.01	5.35 × 10−10
FSD1	1.21	7.00	5.47 × 10−10
LPL	-1.40	-7.03	4.69 × 10−10
DRD1	-1.75	-7.00	5.71 × 10−10
SLC16A11	-1.09	-6.99	6.06 × 10−10
C3	-1.16	-7.09	3.36 × 10−10
SYNDIG1L	-1.46	-6.98	6.25 × 10−10
CADM3-AS1	-1.36	-6.98	6.28 × 10−10
EDN3	-1.74	-6.97	6.45 × 10−10
CCL26	1.42	6.97	6.48 × 10−10
CPO	-1.12	-6.97	6.44 × 10−10
LINC01750	-1.17	-6.97	6.48 × 10−10
ADAMTS9-AS2	-1.04	-6.97	6.66 × 10−10
RTN4RL1	-1.47	-6.97	6.71 × 10−10
HS3ST2	-1.19	-6.97	6.58 × 10−10
LUZP2	-1.80	-6.96	6.86 × 10−10
SIGLEC17P	-1.10	-6.95	7.23 × 10−10
CPB2	-2.28	-6.95	7.28 × 10−10
AC007207.2	-1.94	-6.95	7.28 × 10−10
AC060834.1	-1.52	-6.95	7.20 × 10−10
SEPT14P12	1.17	6.94	7.55 × 10−10
F11-AS1	-1.59	-6.95	7.40 × 10−10
AGER	-1.62	-6.97	6.52 × 10−10
MIR3189	-1.64	-6.94	7.69 × 10−10
RN7SKP51	-1.48	-6.93	8.09 × 10−10
AC004947.2	-1.18	-6.93	8.20 × 10−10
SFTPC	-3.59	-6.96	6.82 × 10−10
WNT11	-1.16	-6.91	9.33 × 10−10
IL33	-1.04	-6.93	8.20 × 10−10
INMT-MINDY4	-1.28	-6.90	9.51 × 10−10
PGBD4P3	-1.06	-6.89	1.02 × 10−09
AC093503.3	-1.03	-6.89	1.03 × 10−09
GFI1B	-1.14	-6.89	1.04 × 10−09
RYR2	-1.04	-6.88	1.07 × 10−09
AC016877.3	1.41	6.88	1.09 × 10−09

DNASE2B	-1.42	-6.87	1.14×10^{-09}
FLRT3	-1.26	-6.90	9.57×10^{-10}
LINC02195	1.34	6.87	1.15×10^{-09}
SLC18A2	-1.23	-6.86	1.19×10^{-09}
ACOD1	1.44	6.87	1.18×10^{-09}
KCNS2	-1.10	-6.86	1.19×10^{-09}
LRRC36	-1.31	-6.86	1.20×10^{-09}
ADRB3	-1.40	-6.86	1.21×10^{-09}
C3orf86	-1.26	-6.85	1.28×10^{-09}
GFRA2	-1.00	-6.84	1.37×10^{-09}
ACSM1	-1.04	-6.82	1.49×10^{-09}
FOXI2	-1.38	-6.82	1.52×10^{-09}
TNNI3K	-1.12	-6.81	1.57×10^{-09}
F13A1	-1.16	-6.84	1.36×10^{-09}
AC006329.1	1.23	6.80	1.71×10^{-09}
MIR1-1HG-AS1	-1.18	-6.80	1.73×10^{-09}
ACSM5	-1.19	-6.79	1.77×10^{-09}
AC092071.1	-1.78	-6.79	1.78×10^{-09}
LINC00469	-1.19	-6.79	1.79×10^{-09}
ELANE	-1.58	-6.79	1.80×10^{-09}
MC5R	-1.27	-6.79	1.81×10^{-09}
CYP4X1	-1.29	-6.80	1.68×10^{-09}
HLX-AS1	-1.04	-6.78	1.95×10^{-09}
AC010271.2	1.08	6.77	1.98×10^{-09}
AL160153.1	-1.06	-6.77	2.00×10^{-09}
MAST1	1.02	6.77	2.07×10^{-09}
ALPL	-1.53	-6.80	1.67×10^{-09}
PTGDS	-1.08	-6.80	1.72×10^{-09}
HCN4	-1.75	-6.75	2.33×10^{-09}
AC004947.1	-1.33	-6.74	2.44×10^{-09}
RORB	-1.51	-6.73	2.59×10^{-09}
SCGB3A1	-2.52	-6.76	2.19×10^{-09}
RNASE1	-1.08	-6.82	1.55×10^{-09}
GPR12	-1.78	-6.72	2.72×10^{-09}
BCHE	-1.10	-6.72	2.71×10^{-09}
DEPDC1-AS1	1.13	6.71	2.82×10^{-09}
AL590226.1	-1.02	-6.71	2.84×10^{-09}
CNR1	-1.23	-6.71	2.79×10^{-09}
PCDH15	-1.64	-6.70	2.93×10^{-09}
AC016769.1	1.39	6.70	3.01×10^{-09}
RGS22	-1.28	-6.69	3.12×10^{-09}
HPGD	-1.56	-6.73	2.47×10^{-09}
AC012085.2	-1.16	-6.69	3.16×10^{-09}
NWD1	-1.86	-6.69	3.16×10^{-09}
SOSTDC1	-1.99	-6.68	3.30×10^{-09}
GKN2	-2.59	-6.67	3.47×10^{-09}
SLC17A3	-1.28	-6.67	3.49×10^{-09}
LCN6	-1.25	-6.67	3.50×10^{-09}
AC145124.1	-1.01	-6.67	3.58×10^{-09}
C6	-1.88	-6.66	3.81×10^{-09}
ZBTB7C	-1.19	-6.67	3.51×10^{-09}
AL355870.1	-1.13	-6.65	3.96×10^{-09}
ENTPD3	-1.06	-6.66	3.81×10^{-09}
SBSN	2.02	6.62	4.66×10^{-09}
C10orf105	-1.07	-6.62	4.77×10^{-09}
AQP4	-2.18	-6.65	4.02×10^{-09}
PIGR	-2.21	-6.69	3.12×10^{-09}
AL031058.1	1.10	6.61	5.04×10^{-09}
KCNK3	-1.25	-6.62	4.74×10^{-09}
CHI3L2	-1.25	-6.61	4.91×10^{-09}
TMEM232	-1.24	-6.59	5.51×10^{-09}
TMEM100	-1.36	-6.60	5.27×10^{-09}
CDH20	-1.60	-6.59	5.55×10^{-09}
AC084759.3	-1.57	-6.58	5.69×10^{-09}
FAM216B	-2.03	-6.58	5.82×10^{-09}
AP003721.1	1.09	6.57	6.08×10^{-09}
GGT6	-1.85	-6.58	5.66×10^{-09}
CYP4F24P	-1.37	-6.57	6.10×10^{-09}
TSLP	-1.11	-6.57	6.15×10^{-09}

AL157895.1	-1.04	-6.56	6.30 × 10−09
MUCL3	-1.96	-6.57	6.20 × 10−09
SFTA2	-1.46	-6.62	4.70 × 10−09
ADGRG2	-1.07	-6.56	6.39 × 10−09
B3GNT8	-1.04	-6.58	5.78 × 10−09
PLA2G4F	-1.31	-6.57	6.10 × 10−09
AC112907.2	1.11	6.54	6.97 × 10−09
AL445493.3	-1.25	-6.54	7.14 × 10−09
HSD17B13	-1.34	-6.54	7.29 × 10−09
ENAM	-1.52	-6.53	7.47 × 10−09
GATA1	-1.01	-6.53	7.49 × 10−09
AC079630.1	-1.63	-6.53	7.46 × 10−09
ABO	-1.25	-6.56	6.34 × 10−09
CCDC141	-1.07	-6.52	7.90 × 10−09
CERNA3	1.00	6.52	7.85 × 10−09
TRHDE	-1.62	-6.52	7.97 × 10−09
NPNT	-1.03	-6.57	6.15 × 10−09
TPSB2	-1.30	-6.54	7.27 × 10−09
SOX6	-1.06	-6.52	8.14 × 10−09
AL109659.2	-1.07	-6.51	8.54 × 10−09
AC090044.1	-1.08	-6.50	8.87 × 10−09
LINC00551	-1.25	-6.48	9.73 × 10−09
STRIP2	1.16	6.49	9.34 × 10−09
ARL9	1.13	6.47	1.05 × 10−08
CDKL2	-1.14	-6.48	9.75 × 10−09
GPM6A	-1.52	-6.46	1.11 × 10−08
DYNLRB2	-1.43	-6.42	1.34 × 10−08
SERPINB10	-1.24	-6.42	1.36 × 10−08
GZMB	1.12	6.44	1.23 × 10−08
MYH2	-1.20	-6.42	1.38 × 10−08
SLC14A1	-1.03	-6.41	1.43 × 10−08
AC010998.3	-1.62	-6.40	1.55 × 10−08
SCUBE2	-1.27	-6.42	1.34 × 10−08
GAS6-AS1	-1.07	-6.40	1.51 × 10−08
BMX	-1.04	-6.38	1.72 × 10−08
CMA1	-1.20	-6.37	1.76 × 10−08
MYMK	-1.07	-6.38	1.70 × 10−08
AC099518.1	1.09	6.37	1.78 × 10−08
AF279873.1	1.05	6.37	1.78 × 10−08
CFTR	-1.59	-6.39	1.59 × 10−08
AC106738.2	-1.45	-6.37	1.81 × 10−08
CD36	-1.03	-6.40	1.55 × 10−08
IYD	-1.72	-6.36	1.83 × 10−08
AP003064.2	-1.21	-6.35	2.00 × 10−08
DNAH6	-1.21	-6.35	2.03 × 10−08
LINC01305	1.41	6.34	2.13 × 10−08
MC2R	-1.17	-6.34	2.04 × 10−08
ANO5	-1.25	-6.34	2.07 × 10−08
RSPO4	-1.34	-6.33	2.15 × 10−08
ZYG11A	1.53	6.33	2.14 × 10−08
SIGLEC6	-1.10	-6.33	2.22 × 10−08
GLP1R	-1.49	-6.32	2.27 × 10−08
COCH	1.43	6.33	2.16 × 10−08
AC133963.1	-1.66	-6.32	2.33 × 10−08
PCSK2	-2.96	-6.31	2.37 × 10−08
SDK2	-1.10	-6.32	2.25 × 10−08
ZNF670-ZNF695	1.03	6.31	2.46 × 10−08
TYRP1	-1.24	-6.31	2.47 × 10−08
CA3	-1.14	-6.30	2.58 × 10−08
MAB21L1	-1.01	-6.29	2.69 × 10−08
PLPPR1	-1.73	-6.30	2.62 × 10−08
ADGRB3	-1.11	-6.27	2.93 × 10−08
AP000526.1	1.17	6.27	3.01 × 10−08
LINC00163	-1.15	-6.26	3.13 × 10−08
ZNF536	-1.22	-6.26	3.20 × 10−08
AL035665.1	-1.12	-6.26	3.24 × 10−08
FAM189A2	-1.05	-6.28	2.86 × 10−08
AC027627.1	1.12	6.27	3.06 × 10−08
AC007671.1	-1.05	-6.25	3.33 × 10−08

GLDC	1.35	6.26	3.22 × 10−08
SILC1	-1.70	-6.24	3.43 × 10−08
TRIM71	-1.70	-6.24	3.51 × 10−08
IFNG	1.38	6.24	3.59 × 10−08
KLF15	-1.11	-6.25	3.28 × 10−08
GCSAML	-1.18	-6.23	3.65 × 10−08
SCGB1A1	-2.96	-6.26	3.20 × 10−08
DNAH9	-2.02	-6.23	3.63 × 10−08
DLEC1	-1.16	-6.23	3.73 × 10−08
CHRM1	-1.36	-6.21	3.99 × 10−08
C5orf38	-1.53	-6.23	3.74 × 10−08
AL355974.2	-1.10	-6.21	4.11 × 10−08
AC044810.2	-1.19	-6.21	4.17 × 10−08
TNN	-1.03	-6.20	4.25 × 10−08
TPPP3	-1.14	-6.25	3.34 × 10−08
SHISA6	-1.37	-6.20	4.36 × 10−08
PGC	-3.11	-6.24	3.45 × 10−08
XCR1	-1.02	-6.20	4.39 × 10−08
LDLRAD4-AS1	-1.00	-6.18	4.79 × 10−08
GGTLC5P	-1.06	-6.19	4.45 × 10−08
SLC34A2	-1.36	-6.32	2.35 × 10−08
NEXMIF	-1.48	-6.16	5.30 × 10−08
CADPS	-1.19	-6.15	5.47 × 10−08
LINC01996	-1.35	-6.15	5.55 × 10−08
C10orf142	-1.17	-6.15	5.56 × 10−08
COL20A1	1.16	6.14	5.78 × 10−08
AC018742.1	-1.27	-6.15	5.70 × 10−08
NXF3	-1.30	-6.14	6.00 × 10−08
LINC01844	-1.30	-6.13	6.02 × 10−08
AL109615.3	1.12	6.14	5.92 × 10−08
TCEAL2	-1.25	-6.13	6.28 × 10−08
ANKRD18B	1.70	6.13	6.28 × 10−08
ACOXL	-1.13	-6.14	5.90 × 10−08
PTPRQ	-1.40	-6.12	6.36 × 10−08
LRRC52-AS1	-1.73	-6.12	6.51 × 10−08
PRSS35	-1.03	-6.11	6.75 × 10−08
TMEM130	-1.36	-6.13	6.23 × 10−08
MASP1	-1.01	-6.11	6.90 × 10−08
AL008733.1	-1.47	-6.10	7.22 × 10−08
HIST1H2BO	1.27	6.10	7.25 × 10−08
SFTPA2	-2.29	-6.24	3.58 × 10−08
COL6A5	-1.23	-6.11	6.90 × 10−08
AC016705.2	-1.41	-6.10	7.27 × 10−08
AC006994.2	-1.06	-6.10	7.24 × 10−08
AL136452.1	-1.38	-6.09	7.71 × 10−08
C4A	-1.07	-6.12	6.44 × 10−08
ZNF385B	-1.55	-6.10	7.05 × 10−08
GATA6-AS1	-1.22	-6.08	7.88 × 10−08
ANGPTL5	-1.06	-6.08	7.99 × 10−08
SERPINA1	-1.23	-6.21	4.06 × 10−08
SCARA5	-1.54	-6.08	7.78 × 10−08
NRAP	-1.35	-6.07	8.36 × 10−08
AC011944.1	-1.22	-6.07	8.40 × 10−08
AL137026.1	-1.26	-6.07	8.49 × 10−08
ADGRF5P1	-1.10	-6.06	8.67 × 10−08
AC018697.1	-1.11	-6.07	8.31 × 10−08
GDF15	-1.14	-6.13	6.16 × 10−08
KIF6	-1.08	-6.07	8.55 × 10−08
KCNJ5	-1.02	-6.08	7.87 × 10−08
ARC	-1.08	-6.06	8.77 × 10−08
SGCG	-1.00	-6.05	9.12 × 10−08
CLDN2	-2.22	-6.07	8.16 × 10−08
LINC02100	1.04	6.05	9.16 × 10−08
ANKRD7	1.24	6.05	9.32 × 10−08
CD1B	-1.35	-6.05	9.30 × 10−08
SNTN	-1.70	-6.05	9.16 × 10−08
PRMT8	-1.79	-6.04	9.78 × 10−08
ENPP7P11	-1.17	-6.03	1.00 × 10−07
TSPAN7	-1.11	-6.07	8.51 × 10−08

ST8SIA6	-1.06	-6.03	1.03 × 10−07
GALNT5	-1.00	-6.07	8.37 × 10−08
AC093772.1	-1.10	-6.02	1.09 × 10−07
AGTR2	-2.25	-6.02	1.10 × 10−07
NSG1	-1.12	-6.00	1.18 × 10−07
MALRD1	-1.25	-5.99	1.23 × 10−07
AC068228.1	1.14	5.99	1.27 × 10−07
NME5	-1.01	-6.00	1.20 × 10−07
AGBL1	-1.11	-6.00	1.20 × 10−07
AQP1	-1.22	-6.09	7.58 × 10−08
IRX2	-1.67	-6.02	1.08 × 10−07
CFAP58	-1.02	-5.98	1.32 × 10−07
AC108156.1	-1.08	-5.98	1.28 × 10−07
IGSF9B	-1.24	-5.99	1.28 × 10−07
CABCOCO1	-1.35	-5.98	1.33 × 10−07
GAL	1.78	5.97	1.36 × 10−07
RGS7BP	-1.20	-5.97	1.38 × 10−07
UBXN10	-1.03	-6.01	1.12 × 10−07
FCGBP	-1.37	-6.03	1.03 × 10−07
TM4SF4	-2.46	-5.97	1.42 × 10−07
RIC3	-1.16	-5.96	1.44 × 10−07
CD1A	-1.72	-5.96	1.43 × 10−07
LINC01612	-1.69	-5.94	1.58 × 10−07
AC131009.2	1.27	5.94	1.57 × 10−07
KNDC1	-1.40	-5.96	1.43 × 10−07
KIR2DL4	1.39	5.93	1.66 × 10−07
CNTFR	-1.37	-5.93	1.69 × 10−07
SFTPA1	-2.33	-6.06	8.60 × 10−08
RGS20	1.24	5.93	1.68 × 10−07
FO681492.1	-1.09	-5.93	1.72 × 10−07
C15orf48	1.17	5.98	1.29 × 10−07
SEC14L3	-1.53	-5.91	1.86 × 10−07
GNG4	1.64	5.92	1.73 × 10−07
LINC00355	1.87	5.90	1.97 × 10−07
KCNJ15	-1.27	-5.95	1.49 × 10−07
P2RY12	-1.08	-5.89	2.01 × 10−07
C14orf180	-1.15	-5.89	2.02 × 10−07
HMGCLL1	-1.00	-5.89	2.06 × 10−07
ANKRD34C	-1.06	-5.94	1.63 × 10−07
PCDH11X	-1.10	-5.88	2.11 × 10−07
OCA2	-1.46	-5.88	2.17 × 10−07
ELN-AS1	-1.02	-5.88	2.13 × 10−07
C5orf49	-1.25	-5.89	2.06 × 10−07
HIST1H2BG	1.49	5.88	2.20 × 10−07
GRIN2A	-1.78	-5.87	2.30 × 10−07
VIPR1-AS1	-1.02	-5.87	2.30 × 10−07
OR51E1	1.25	5.86	2.38 × 10−07
GPC3	-1.06	-5.89	2.02 × 10−07
PIFO	-1.03	-5.88	2.17 × 10−07
MRC1	-1.02	-5.92	1.81 × 10−07
AL354861.3	-1.08	-5.86	2.36 × 10−07
APOD	-1.34	-5.89	2.01 × 10−07
HIST1H2AE	1.49	5.84	2.65 × 10−07
CLIC5	-1.11	-5.88	2.11 × 10−07
CLEC9A	-1.01	-5.83	2.70 × 10−07
MIR137HG	1.47	5.83	2.72 × 10−07
LINC02471	-1.69	-5.83	2.74 × 10−07
AQP3	-1.20	-5.96	1.48 × 10−07
AC091173.1	1.36	5.83	2.74 × 10−07
PPP1R1B	-1.65	-5.87	2.23 × 10−07
LINC01214	1.56	5.81	3.04 × 10−07
C1orf87	-1.85	-5.81	3.04 × 10−07
LINC02657	1.39	5.81	3.06 × 10−07
GPD1	-1.09	-5.82	2.84 × 10−07
LINC02587	-1.03	-5.80	3.17 × 10−07
TERT	1.23	5.80	3.13 × 10−07
OR5BA1P	-1.04	-5.80	3.21 × 10−07
LGR6	-1.18	-5.82	2.94 × 10−07
CXCL10	1.12	5.85	2.43 × 10−07

LINC01625	-1.01	-5.80	3.16 × 10−07
IGF2BP3	1.58	5.81	2.95 × 10−07
DACH2	-1.25	-5.79	3.38 × 10−07
AQP7	-1.33	-5.80	3.19 × 10−07
CYP2A6	-1.45	-5.78	3.45 × 10−07
CEACAM6	-1.36	-5.95	1.54 × 10−07
CDIPTOSP	1.26	5.78	3.52 × 10−07
HIST1H1B	1.38	5.77	3.63 × 10−07
ANO4	-1.23	-5.77	3.65 × 10−07
COL21A1	-1.04	-5.80	3.18 × 10−07
HIST1H2BJ	1.04	5.77	3.60 × 10−07
AC089983.1	1.41	5.76	3.80 × 10−07
OVCH2	-1.28	-5.75	4.01 × 10−07
IHH	-1.53	-5.75	4.13 × 10−07
PCARE	-1.40	-5.74	4.16 × 10−07
TRPV6	-1.42	-5.75	4.13 × 10−07
SHISA3	-1.80	-5.75	4.12 × 10−07
NRG3	-1.11	-5.72	4.61 × 10−07
AC073862.5	-1.17	-5.72	4.66 × 10−07
NPY5R	-1.07	-5.73	4.43 × 10−07
MIR4697HG	-1.29	-5.74	4.35 × 10−07
CYP4Z1	-1.22	-5.71	4.90 × 10−07
UGT2B15	-1.93	-5.71	4.91 × 10−07
HIST1H3F	1.25	5.72	4.78 × 10−07
AC122710.2	1.02	5.71	4.97 × 10−07
VGF	1.47	5.71	4.90 × 10−07
SLC22A31	-1.33	-5.79	3.36 × 10−07
HIST2H2AB	1.13	5.70	5.10 × 10−07
AP004608.1	-1.97	-5.71	4.83 × 10−07
CYP4F12	-1.36	-5.70	5.14 × 10−07
FAM81B	-1.34	-5.71	5.01 × 10−07
AC020928.2	1.29	5.69	5.37 × 10−07
AC013264.1	-1.33	-5.69	5.53 × 10−07
MAS1L	-1.08	-5.70	5.12 × 10−07
KCNG3	1.23	5.68	5.57 × 10−07
RPS29P11	-1.24	-5.68	5.63 × 10−07
ITIH2	-1.60	-5.68	5.63 × 10−07
BAAT	-1.57	-5.68	5.59 × 10−07
KLC3	1.06	5.70	5.20 × 10−07
ARSEP1	-1.51	-5.67	5.93 × 10−07
CFAP57	-1.10	-5.69	5.45 × 10−07
AC016205.1	1.18	5.67	5.85 × 10−07
PDZRN4	-1.15	-5.67	5.97 × 10−07
CDH19	-1.31	-5.67	6.04 × 10−07
HOPX	-1.19	-5.78	3.56 × 10−07
CNTN3	-1.21	-5.66	6.18 × 10−07
KIF12	-1.31	-5.68	5.63 × 10−07
AC105254.2	-1.22	-5.65	6.57 × 10−07
CAPN6	-1.78	-5.65	6.51 × 10−07
LINC01550	-1.03	-5.65	6.54 × 10−07
PCMTD1P3	1.16	5.64	6.93 × 10−07
LINC00443	-1.08	-5.65	6.67 × 10−07
KIAA0408	-1.15	-5.63	7.07 × 10−07
CBLN4	-1.13	-5.63	7.08 × 10−07
DMBT1	-2.02	-5.68	5.77 × 10−07
ODAM	-1.36	-5.60	8.42 × 10−07
AC108215.1	-1.30	-5.60	8.11 × 10−07
RAB3C	-1.12	-5.59	8.71 × 10−07
AC129507.3	-1.12	-5.58	8.94 × 10−07
IL5RA	-1.15	-5.59	8.71 × 10−07
AC115099.1	-1.14	-5.58	9.07 × 10−07
AC008268.1	-2.05	-5.58	8.96 × 10−07
FP325330.3	1.22	5.59	8.67 × 10−07
HAGLR	-1.13	-5.64	6.78 × 10−07
IGSF10	-1.02	-5.60	8.38 × 10−07
RN7SL8P	-1.32	-5.56	1.01 × 10−06
FOSB	-1.43	-5.63	7.29 × 10−07
SYNPR-AS1	-1.31	-5.55	1.03 × 10−06
SULT1E1	-1.24	-5.54	1.08 × 10−06

SULT1C2	-1.23	-5.59	8.75 × 10−07
RS1	-1.10	-5.54	1.11 × 10−06
CAPN9	-1.34	-5.57	9.75 × 10−07
AC010998.1	-1.18	-5.54	1.11 × 10−06
LINC02446	1.25	5.53	1.13 × 10−06
AC024940.2	1.11	5.53	1.17 × 10−06
SLC46A2	-1.29	-5.54	1.08 × 10−06
LMO3	-1.22	-5.61	7.73 × 10−07
AC096734.1	1.02	5.52	1.18 × 10−06
AL031777.1	1.09	5.52	1.20 × 10−06
GADL1	-1.13	-5.52	1.21 × 10−06
CHIT1	-1.44	-5.58	8.94 × 10−07
LRRC18	-1.24	-5.51	1.25 × 10−06
HMGCS2	-2.00	-5.51	1.27 × 10−06
SLC6A4	-1.31	-5.51	1.28 × 10−06
AL117329.1	1.51	5.50	1.30 × 10−06
SORCS2	-1.12	-5.55	1.03 × 10−06
FOXD3-AS1	1.51	5.50	1.31 × 10−06
TMEM114	-1.04	-5.52	1.22 × 10−06
STEAP1B	1.04	5.50	1.32 × 10−06
CCL8	1.03	5.52	1.23 × 10−06
RXRG	-1.25	-5.49	1.38 × 10−06
KRT27	-1.16	-5.48	1.43 × 10−06
PRODH	-1.39	-5.54	1.09 × 10−06
RAD17P1	-1.24	-5.48	1.46 × 10−06
SULT1C2P1	-1.17	-5.47	1.49 × 10−06
HIST1H4D	1.17	5.47	1.53 × 10−06
CNGA4	-1.16	-5.47	1.53 × 10−06
LINC00525	1.19	5.47	1.54 × 10−06
TREH	-1.02	-5.47	1.54 × 10−06
SLPI	-1.35	-5.58	9.17 × 10−07
CFAP61	-1.03	-5.46	1.57 × 10−06
AADAC	-1.67	-5.47	1.55 × 10−06
COLCA1	-1.11	-5.50	1.32 × 10−06
HIST1H2BH	1.33	5.45	1.64 × 10−06
RGS6	-1.41	-5.45	1.64 × 10−06
HIST1H2AM	1.06	5.45	1.66 × 10−06
ALDOB	-1.37	-5.44	1.71 × 10−06
CA8	-1.34	-5.46	1.58 × 10−06
ITGB1-DT	1.15	5.43	1.84 × 10−06
KCNQ5-IT1	1.06	5.44	1.74 × 10−06
LINC00570	-1.07	-5.40	2.12 × 10−06
AC131097.3	1.18	5.39	2.20 × 10−06
AC005592.1	-1.03	-5.38	2.25 × 10−06
PI16	-1.21	-5.38	2.33 × 10−06
GSTA3	-1.24	-5.37	2.40 × 10−06
GSDMC	1.09	5.40	2.07 × 10−06
AL021328.1	-1.13	-5.37	2.43 × 10−06
LHFPL3	-1.44	-5.37	2.44 × 10−06
ATOH8	-1.18	-5.42	1.87 × 10−06
AC073862.2	-1.09	-5.36	2.55 × 10−06
ABCC12	-1.15	-5.36	2.57 × 10−06
CDH18	1.59	5.36	2.54 × 10−06
AL049830.3	1.07	5.35	2.62 × 10−06
GBX2	1.29	5.35	2.61 × 10−06
ARSE	-1.35	-5.40	2.07 × 10−06
PKHD1	-1.43	-5.35	2.59 × 10−06
ZDHHC11B	-1.21	-5.38	2.34 × 10−06
VWA3A	-1.39	-5.35	2.65 × 10−06
CFAP65	-1.34	-5.33	2.84 × 10−06
TWIST1	1.15	5.33	2.83 × 10−06
RETNLB	1.15	5.32	3.01 × 10−06
SORCS1	-1.22	-5.31	3.23 × 10−06
AL135999.3	-1.57	-5.32	3.04 × 10−06
ADRA2A	-1.11	-5.35	2.68 × 10−06
CDKN2A	1.35	5.35	2.63 × 10−06
SLITRK3	-1.18	-5.29	3.41 × 10−06
AC005725.1	-1.15	-5.29	3.41 × 10−06
NPY1R	-1.06	-5.30	3.34 × 10−06

AC010627.1	-1.04	-5.28	3.59 × 10−06
C20orf141	1.04	5.31	3.13 × 10−06
NMUR2	-1.14	-5.28	3.63 × 10−06
LRRC55	-1.20	-5.28	3.63 × 10−06
LINC01234	1.71	5.27	3.82 × 10−06
MS4A8	-1.95	-5.28	3.66 × 10−06
STEAP2-AS1	1.01	5.26	3.97 × 10−06
HIST1H1E	1.08	5.26	3.91 × 10−06
AC079467.1	-1.47	-5.26	3.95 × 10−06
CLUL1	-1.01	-5.28	3.71 × 10−06
HIST1H2AL	1.05	5.26	3.99 × 10−06
LINC01535	1.04	5.27	3.90 × 10−06
ALOX15B	-1.19	-5.34	2.75 × 10−06
ERICH3	-1.76	-5.27	3.88 × 10−06
CFAP46	-1.28	-5.26	3.91 × 10−06
CES1	-1.22	-5.34	2.73 × 10−06
SMIM6	-1.02	-5.28	3.70 × 10−06
PCDHAC2	-1.01	-5.27	3.89 × 10−06
BMP3	-1.32	-5.30	3.34 × 10−06
APOH	-1.73	-5.24	4.29 × 10−06
STAC2	-1.14	-5.23	4.56 × 10−06
SCN1A	-1.36	-5.23	4.47 × 10−06
AL591468.1	1.01	5.22	4.85 × 10−06
TEX19	1.27	5.21	4.90 × 10−06
CCDC198	-1.85	-5.21	4.88 × 10−06
VEPH1	-1.07	-5.26	3.97 × 10−06
DNAH17-AS1	1.07	5.22	4.83 × 10−06
AC087588.2	1.03	5.21	5.08 × 10−06
LINC00332	-1.10	-5.23	4.52 × 10−06
SRD5A2	-1.49	-5.21	4.93 × 10−06
AC005550.2	-1.06	-5.22	4.84 × 10−06
C4orf19	-1.11	-5.23	4.48 × 10−06
HHIP-AS1	-1.21	-5.22	4.85 × 10−06
APCDD1L	1.33	5.21	5.03 × 10−06
DNAI2	-1.44	-5.18	5.65 × 10−06
LINC00629	1.03	5.17	5.93 × 10−06
CRYM	-1.37	-5.22	4.68 × 10−06
DUOX2	-1.32	-5.20	5.25 × 10−06
ABCC8	-1.39	-5.16	6.08 × 10−06
HIST1H1D	1.29	5.16	6.19 × 10−06
AGR3	-1.43	-5.23	4.50 × 10−06
LANCL3	-1.07	-5.16	6.07 × 10−06
XKR4	-1.08	-5.15	6.52 × 10−06
AC093523.1	-1.00	-5.17	6.00 × 10−06
BRD9P2	-1.04	-5.14	6.94 × 10−06
FREM2	-1.15	-5.19	5.48 × 10−06
ELDR	1.06	5.13	7.00 × 10−06
DIRAS3	-1.01	-5.14	6.66 × 10−06
AC018781.1	1.12	5.12	7.37 × 10−06
AC120498.2	-1.10	-5.12	7.41 × 10−06
DLX2	1.13	5.13	7.19 × 10−06
NR5A1	1.08	5.12	7.58 × 10−06
OR2B6	1.04	5.11	7.66 × 10−06
HIF3A	-1.21	-5.16	6.18 × 10−06
LINC02323	1.06	5.11	7.60 × 10−06
SCNN1G	-1.08	-5.16	6.12 × 10−06
14-Sep	1.05	5.14	6.92 × 10−06
LINC02568	-1.19	-5.10	8.06 × 10−06
AC007906.2	-1.30	-5.12	7.29 × 10−06
GRM5-AS1	1.01	5.09	8.55 × 10−06
AL645608.8	1.05	5.08	8.78 × 10−06
HHLA2	-2.08	-5.10	7.97 × 10−06
C9orf135	-1.51	-5.08	8.89 × 10−06
ADGRA1	-1.16	-5.07	9.10 × 10−06
AC012213.1	1.38	5.07	9.15 × 10−06
SNTG2	-1.00	-5.06	9.69 × 10−06
GREB1L	1.12	5.09	8.53 × 10−06
ST6GALNAC1	-1.11	-5.15	6.47 × 10−06
TSPAN8	-1.49	-5.13	7.05 × 10−06

PTCHD1	-1.20	-5.06	9.80 × 10−06
LUCAT1	1.24	5.07	9.38 × 10−06
HPR	-1.09	-5.04	1.06 × 10−05
AL139412.1	1.07	5.04	1.05 × 10−05
SH2D5	1.20	5.04	1.07 × 10−05
CA4	-1.36	-5.05	1.03 × 10−05
CCL7	1.16	5.04	1.05 × 10−05
HCN1	-1.55	-5.03	1.10 × 10−05
GGTLC2	-1.09	-5.03	1.11 × 10−05
FAM222A-AS1	1.03	5.03	1.10 × 10−05
LINC01671	-1.22	-5.05	1.01 × 10−05
AC010735.2	1.05	5.03	1.11 × 10−05
C2orf73	-1.16	-5.02	1.13 × 10−05
DIO3	-1.06	-5.03	1.12 × 10−05
SYNGR4	1.03	5.02	1.13 × 10−05
UCN3	-1.70	-5.02	1.17 × 10−05
FOSL1	1.01	5.06	9.69 × 10−06
AL390778.2	-1.10	-5.01	1.22 × 10−05
CCDC60	-1.37	-5.01	1.23 × 10−05
ACE2	-1.10	-5.03	1.09 × 10−05
HAS3	-1.06	-5.07	9.31 × 10−06
SHOC1	1.02	4.99	1.29 × 10−05
HIST1H3G	1.22	4.96	1.47 × 10−05
AZU1	-1.10	-4.96	1.49 × 10−05
C20orf85	-2.00	-4.96	1.47 × 10−05
LINC02555	-1.47	-4.95	1.55 × 10−05
CXCL11	1.05	4.99	1.32 × 10−05
C8B	-1.54	-4.94	1.60 × 10−05
TEX48	1.04	4.93	1.68 × 10−05
SRGAP3-AS2	-1.79	-4.93	1.71 × 10−05
HIST1H3C	1.08	4.92	1.74 × 10−05
CA10	-1.95	-4.92	1.75 × 10−05
LRRC52	-1.28	-4.92	1.80 × 10−05
MEGF11	-1.25	-4.93	1.71 × 10−05
CPA4	1.23	4.91	1.82 × 10−05
AC017048.3	1.03	4.91	1.83 × 10−05
WT1-AS	1.19	4.90	1.93 × 10−05
LINC02616	1.41	4.90	1.89 × 10−05
FGFR3	-1.09	-4.97	1.42 × 10−05
PSORS1C3	-1.41	-4.89	2.04 × 10−05
AC026992.2	-1.01	-4.89	2.04 × 10−05
LINC01224	1.49	4.89	2.01 × 10−05
AC243967.2	-1.01	-4.88	2.10 × 10−05
HOXA11-AS	1.27	4.87	2.16 × 10−05
NR0B2	-1.52	-4.89	2.01 × 10−05
NKX2-1	-1.07	-5.01	1.19 × 10−05
STMND1	-1.30	-4.88	2.08 × 10−05
SLC16A12	-1.14	-4.88	2.09 × 10−05
CAPN13	-1.14	-4.94	1.64 × 10−05
CHRM2	-1.10	-4.86	2.28 × 10−05
FAM83A	1.10	4.98	1.39 × 10−05
KLHDC7A	-1.06	-4.90	1.90 × 10−05
LY6K	1.82	4.88	2.10 × 10−05
HBB	-1.14	-4.92	1.77 × 10−05
AC012317.1	-1.13	-4.87	2.21 × 10−05
SLC10A2	-1.30	-4.85	2.39 × 10−05
NKX1-2	1.73	4.83	2.54 × 10−05
HIST1H2BF	1.13	4.82	2.66 × 10−05
CHST9	-1.83	-4.83	2.54 × 10−05
HIST1H2AD	1.20	4.82	2.66 × 10−05
PCDH10	-1.01	-4.82	2.65 × 10−05
AQP2	-1.28	-4.82	2.72 × 10−05
TNNT1	1.56	4.87	2.16 × 10−05
NT5C1A	-1.19	-4.80	2.87 × 10−05
AC092691.1	-1.04	-4.82	2.71 × 10−05
AC025259.3	-1.02	-4.81	2.82 × 10−05
AL512328.1	-1.03	-4.79	3.05 × 10−05
GAS2L2	-1.26	-4.80	2.91 × 10−05
GLYATL1P4	-1.10	-4.79	3.05 × 10−05

AC004832.1	-1.14	-4.78	3.13 × 10−05
SP8	1.77	4.78	3.14 × 10−05
LILRP2	1.06	4.77	3.32 × 10−05
GLP2R	-1.13	-4.77	3.34 × 10−05
SPINK5	-1.22	-4.83	2.60 × 10−05
TPRG1LP1	-1.06	-4.76	3.40 × 10−05
TMEM212	-1.41	-4.76	3.43 × 10−05
LINC01804	1.27	4.76	3.37 × 10−05
LHFPL3-AS1	-1.00	-4.77	3.25 × 10−05
MS4A15	-1.65	-4.78	3.10 × 10−05
HSD17B2	-1.40	-4.77	3.33 × 10−05
PLA2G3	-1.34	-4.76	3.47 × 10−05
STMN2	-1.20	-4.75	3.61 × 10−05
CFAP77	-1.29	-4.75	3.59 × 10−05
IL13RA2	-1.10	-4.75	3.52 × 10−05
SNX18P7	1.09	4.73	3.82 × 10−05
SLC5A7	-1.17	-4.73	3.88 × 10−05
AC129507.2	-1.04	-4.73	3.82 × 10−05
ERN2	-1.75	-4.77	3.36 × 10−05
DPP10	-1.54	-4.74	3.78 × 10−05
DUSP5P1	1.12	4.71	4.16 × 10−05
FAM83A-AS1	1.03	4.74	3.65 × 10−05
CALML3-AS1	1.12	4.69	4.55 × 10−05
ANKRD66	-1.36	-4.69	4.65 × 10−05
ZSCAN4	-1.23	-4.68	4.71 × 10−05
C1orf158	-1.47	-4.68	4.70 × 10−05
AC012213.4	1.22	4.69	4.54 × 10−05
FOXD3	1.21	4.66	5.06 × 10−05
CCL17	-1.07	-4.69	4.63 × 10−05
FAM3D	-1.22	-4.69	4.66 × 10−05
MAGEE2	-1.11	-4.65	5.39 × 10−05
SOHLH1	1.12	4.67	4.98 × 10−05
C1QL1	1.02	4.65	5.46 × 10−05
LINC01819	-1.01	-4.65	5.44 × 10−05
LTF	-1.40	-4.75	3.63 × 10−05
HNF1A-AS1	-1.55	-4.64	5.53 × 10−05
ESRRG	-1.09	-4.65	5.37 × 10−05
CYCSP6	1.33	4.63	5.75 × 10−05
LGR5	-1.12	-4.65	5.47 × 10−05
RERGL	-1.29	-4.63	5.90 × 10−05
LRAT	-1.04	-4.66	5.23 × 10−05
FABP4	-1.13	-4.65	5.34 × 10−05
C1orf61	1.26	4.63	5.78 × 10−05
C12orf56	1.55	4.62	6.12 × 10−05
LRRC71	-1.17	-4.61	6.34 × 10−05
LINC01116	1.08	4.62	6.02 × 10−05
TRIM63	-1.10	-4.59	6.71 × 10−05
MST1L	-1.11	-4.64	5.52 × 10−05
AQP5	-1.76	-4.66	5.23 × 10−05
PRND	-1.10	-4.58	6.97 × 10−05
NTNG1	-1.11	-4.59	6.84 × 10−05
RIMS4	-1.28	-4.58	7.17 × 10−05
IL36RN	1.56	4.58	6.99 × 10−05
ISM2	1.11	4.57	7.48 × 10−05
SPOCK3	-1.41	-4.58	7.16 × 10−05
LINC01644	-1.25	-4.57	7.43 × 10−05
ADRB1	-1.02	-4.59	6.80 × 10−05
RIMS2	1.46	4.56	7.68 × 10−05
TEKT4	-1.02	-4.55	7.88 × 10−05
AL161618.1	-1.04	-4.55	8.01 × 10−05
GSTA1	-1.48	-4.60	6.65 × 10−05
AC226118.1	-1.04	-4.55	8.06 × 10−05
CPA6	-1.23	-4.54	8.19 × 10−05
MMP28	-1.12	-4.61	6.30 × 10−05
CXCL17	-1.00	-4.70	4.43 × 10−05
CLDN8	-1.50	-4.54	8.27 × 10−05
AC005515.1	1.05	4.53	8.78 × 10−05
LINC02577	1.53	4.53	8.66 × 10−05
PLA2G4E	-1.20	-4.54	8.23 × 10−05

CGREF1	1.00	4.55	7.93 × 10−05
C6orf222	-1.31	-4.51	9.31 × 10−05
IL17C	1.06	4.49	1.00 × 10−04
TEPP	-1.14	-4.50	9.58 × 10−05
AC078820.1	1.08	4.49	0.000102636
AL357093.2	-1.23	-4.49	0.000101472
IGF2BP1	1.81	4.49	9.97 × 10−05
CYP3A5	-1.05	-4.52	8.93 × 10−05
ACTL8	1.23	4.48	0.000102807
EFCAB1	-1.26	-4.49	9.88 × 10−05
HBA2	-1.02	-4.51	9.16 × 10−05
NELL1	-1.99	-4.47	0.000110036
CSAG3	1.38	4.46	0.000111563
GRP	-1.20	-4.46	0.000115359
SERTM1	-1.24	-4.44	0.000120805
AC090809.1	1.29	4.51	9.43 × 10−05
AL713965.1	-1.01	-4.44	0.000121031
C6orf118	-1.37	-4.44	0.000121927
HOXA11	1.30	4.43	0.000126575
FAM135B	-1.04	-4.42	0.000129891
AP003481.1	-1.02	-4.45	0.000115683
ELFN1-AS1	1.18	4.42	0.000133478
B4GALNT4	1.16	4.47	0.000110033
ADGB	-1.23	-4.40	0.000140546
TPTEP1	-1.11	-4.45	0.000117502
AC106875.1	1.23	4.43	0.000124469
LINC02133	-1.13	-4.39	0.00014636
LINC00964	-1.01	-4.41	0.000137954
CFAP47	-1.17	-4.40	0.000141553
TOX3	-1.13	-4.48	0.000104121
BMP7	-1.07	-4.43	0.000127718
AC078923.1	1.11	4.39	0.000150189
RNF128	-1.02	-4.46	0.000112484
SHISA2	-1.08	-4.44	0.000122417
AC027281.1	-1.04	-4.37	0.000161192
MROH9	-1.04	-4.33	0.000182385
CSTP1	-1.07	-4.33	0.000185075
RAB3B	1.22	4.37	0.000157922
RMST	-1.08	-4.32	0.000192243
PAPPA2	-1.06	-4.33	0.000188549
SH3GL3	-1.06	-4.31	0.000198548
CHRM3	-1.01	-4.34	0.000179245
WT1	1.18	4.32	0.000193039
KCNB1	-1.05	-4.31	0.000199852
CSAG1	1.69	4.30	0.000208666
MAGEA3	2.13	4.30	0.000208682
ZIC2	1.55	4.31	0.00020387
G2E3-AS1	1.17	4.34	0.000177119
TEKT1	-1.48	-4.31	0.000198575
AC124067.4	-1.14	-4.29	0.000215749
AC091806.1	-1.04	-4.29	0.000214278
MYBPHL	-1.33	-4.31	0.000197667
HNF4A	-1.56	-4.30	0.000208214
MMP12	1.14	4.38	0.000152879
AP002478.1	1.11	4.27	0.000230687
ADH1C	-1.35	-4.32	0.00019432
LINC02489	-1.17	-4.28	0.000225915
AC133785.1	1.17	4.27	0.000230347
OLIG1	-1.06	-4.27	0.000235917
TRHD × 10−AS1	-1.03	-4.26	0.000241351
SERPIND1	-1.43	-4.30	0.000208943
AF279873.3	1.03	4.34	0.000178848
TPSD1	-1.23	-4.27	0.000231515
HOXA10	1.33	4.26	0.000237509
MKRN9P	1.01	4.25	0.000254431
DPPA3P2	-1.00	-4.23	0.000270385
GFY	1.16	4.24	0.00026622
GAL3ST2	1.03	4.23	0.000274656
LRRC74B	-1.15	-4.22	0.000280471

RIMBP2	-1.30	-4.22	0.000285547
CST2	-1.05	-4.23	0.000270458
SFRP5	-1.18	-4.21	0.000292841
DUSP9	1.22	4.22	0.000280687
MAPK4	-1.30	-4.22	0.000281317
POU4F1	1.33	4.20	0.000301311
LHX1-DT	1.17	4.22	0.000285202
TMEM213	-1.22	-4.23	0.000276147
CFAP100	-1.00	-4.20	0.000299491
LINC02122	-1.19	-4.19	0.000315844
FOXQ1	-1.17	-4.27	0.000229382
CSAG2	1.34	4.22	0.000285285
CYP2C18	-1.21	-4.21	0.000290268
PHACTR3	-1.19	-4.24	0.000257347
FYB2	-1.10	-4.20	0.000300142
ITPRID1	-1.54	-4.18	0.000323764
AC025154.2	-1.38	-4.19	0.000317708
AC131532.1	1.19	4.19	0.000316212
ASTN1	-1.03	-4.16	0.000352007
AC010789.1	1.19	4.16	0.000353671
AP005435.1	-1.14	-4.14	0.000369115
ADGRD1-AS1	-1.33	-4.17	0.000331986
GCNT3	-1.07	-4.25	0.000255474
PITX1	1.17	4.21	0.000289074
LINC02532	-1.10	-4.13	0.000386017
APOBEC4	-1.23	-4.13	0.000392716
PRIMA1	-1.01	-4.13	0.000394199
MSLN	-1.67	-4.28	0.000225934
FGF5	1.10	4.11	0.000418903
TTC29	-1.22	-4.10	0.000425965
DLL3	1.37	4.11	0.000416423
KHDC1L	1.11	4.09	0.000446716
PLD5	-1.23	-4.13	0.00039475
AC078925.4	-1.26	-4.13	0.000382802
USH1G	1.11	4.08	0.000468651
CYP4F26P	1.05	4.07	0.00047496
LGALS4	-1.49	-4.12	0.000401755
C11orf97	-1.14	-4.07	0.000486512
SP9	1.25	4.10	0.000433649
CDH16	-1.19	-4.06	0.00050256
TUBA4B	-1.05	-4.07	0.00048261
KIAA2012	-1.05	-4.05	0.000515504
CWH43	-1.23	-4.04	0.000535445
CAPSL	-1.26	-4.05	0.000510525
SEMA3E	-1.12	-4.10	0.000428744
SPAG6	-1.34	-4.07	0.00048261
ORM1	-1.16	-4.08	0.000460547
CDHR4	-1.20	-4.05	0.000525479
LINC01765	-1.02	-4.02	0.000583401
AC010595.1	1.07	4.03	0.000560486
ENPP3	-1.12	-4.07	0.00048279
ALX1	1.14	3.99	0.000629981
ORM2	-1.15	-4.03	0.000561236
ALDH3A1	-1.40	-4.06	0.000494925
HP	-1.46	-4.08	0.000473428
SYT8	-1.10	-4.04	0.000539054
HHATL	-1.15	-3.96	0.000698881
ZIC5	1.28	3.96	0.000720805
BEX1	-1.04	-3.96	0.000721152
YBX2	1.10	4.00	0.000618998
C11orf88	-1.12	-3.96	0.000703233
COL11A1	1.23	4.06	0.000492737
AC011632.1	1.10	3.93	0.000776848
MAGEA6	1.90	3.93	0.000802098
MIR663AHG	-1.26	-3.95	0.000748749
BCAS1	-1.06	-4.01	0.000590347
AC004836.1	-1.10	-3.91	0.000842677
ABCA13	-1.01	-3.97	0.00068255
IRX6	-1.18	-3.93	0.00080206

AC104031.1	-1.01	-3.89	0.000890967
SPINK1	-1.54	-4.01	0.000600466
LINC00973	1.35	3.87	0.000962957
GSTA2	-1.35	-3.89	0.000890982
AC023421.1	-1.63	-3.86	0.001007654
CYP2F1	-1.09	-3.84	0.001072614
AOC1	-1.22	-3.94	0.000757899
DUOXA2	-1.03	-3.85	0.001053587
LINC01257	1.01	3.86	0.000996413
ECEL1P2	-1.01	-3.84	0.001080581
ADGRF4	1.11	3.86	0.001010979
LHX1	1.21	3.80	0.001244546
SMIM31	-1.31	-3.83	0.001104861
EPHA6	1.02	3.79	0.001277231
PIH1D3	-1.11	-3.77	0.001336207
CRABP1	1.33	3.79	0.001290595
DPP10-AS1	-1.21	-3.78	0.001325788
AC069277.1	1.02	3.77	0.001360599
AC139749.1	-1.08	-3.77	0.001375147
LINC01833	1.31	3.75	0.001466234
NR1H4	-1.05	-3.73	0.001545879
AL133320.1	-1.02	-3.73	0.001538086
SERTM2	-1.04	-3.71	0.001663707
CES1P1	-1.02	-3.71	0.001659211
CALML3	1.19	3.70	0.001703516
AC027288.1	-1.13	-3.71	0.001673953
DSCR8	1.18	3.70	0.001684512
LINC01108	-1.15	-3.67	0.001866007
C1orf141	-1.02	-3.67	0.001894784
NEFL	-1.10	-3.67	0.001901692
LINC02241	1.08	3.71	0.001663707
AL160271.1	1.12	3.73	0.001524866
DRAIC	-1.21	-3.70	0.001697169
ITLN2	-1.15	-3.65	0.002027936
AC061975.6	1.13	3.64	0.002095266
AZGP1	-1.29	-3.75	0.001434001
PLA2G12B	-1.25	-3.65	0.001988561
POPDC3	1.16	3.64	0.002069184
PIP	-1.22	-3.63	0.002160426
SLC44A5	-1.01	-3.70	0.001727356
AC008870.5	-1.04	-3.60	0.002353969
AC103702.2	1.15	3.59	0.002479805
LDLRAD1	-1.03	-3.64	0.002109249
CHRNA9	1.29	3.58	0.002514906
S100A7	1.21	3.55	0.002754155
MUC21	-1.31	-3.64	0.002064773
PCSK1	1.22	3.55	0.002788205
CTAG2	1.19	3.49	0.003364975
MYEOV	1.11	3.55	0.002743192
SLC13A2	-1.40	-3.46	0.00365756
ANKS4B	-1.27	-3.45	0.003720041
SLC6A15	1.14	3.44	0.003937918
PWWP3B	-1.04	-3.47	0.003556151
PRAME	1.54	3.49	0.003345917
MAGEA11	1.06	3.46	0.003669708
AP005233.2	1.06	3.41	0.004237505
PAX7	-1.62	-3.41	0.004248404
AMBP	-1.11	-3.45	0.003801915
CACNG6	-1.08	-3.41	0.004279644
GABRB3	-1.02	-3.43	0.004008946
NFE4	1.08	3.35	0.005123645
STOML3	-1.05	-3.33	0.005363812
HNF1A	-1.11	-3.33	0.005465114
HHIPL2	1.12	3.36	0.004884164
GYG2P1	-1.22	-3.28	0.006186347
DUSP13	1.11	3.30	0.005892981
LINC01446	1.12	3.28	0.006200462
SLC5A1	-1.15	-3.33	0.005327551
HOXA13	1.05	3.26	0.006550171

AC011297.1	-1.22	-3.26	0.006711992
SERPINA4	-1.26	-3.17	0.008563421
FAR2P1	1.21	3.14	0.009248678
GPR158	1.00	3.17	0.008570117
LIN28B	1.12	3.17	0.00849573
MAGEC2	1.27	3.14	0.009401317
SCGB2A1	-1.01	-3.14	0.009248249
VSIG1	-1.09	-3.23	0.007232788
LINC01419	1.10	3.16	0.008859269
B3GNT6	-1.16	-3.19	0.008110565
SLC14A2	-1.14	-3.10	0.010313892
TMEM59L	-1.01	-3.19	0.008183556
CNMD	-1.25	-3.07	0.011402859
TSPAN19	-1.06	-3.08	0.011125151
SLC5A8	-1.12	-3.07	0.011351635
CST1	-1.09	-3.12	0.00997865
KLK11	-1.10	-3.12	0.009748285
DACT2	-1.04	-3.02	0.013090413
REG1A	-1.04	-2.95	0.015603231
IL37	-1.12	-2.94	0.016012936
SYT13	-1.26	-2.95	0.015720619
MAGEA12	1.25	2.88	0.018566865
COL25A1	-1.08	-2.88	0.018590899
EREG	1.06	2.90	0.017612162
C1QL2	-1.02	-2.77	0.024719272
GLB1L3	-1.13	-2.81	0.022142152
BPIFB2	-1.21	-2.74	0.026453855
MUC5B	-1.21	-2.84	0.020601021

Table S3. The DEGs identified between mitophagy groups in TCGA-LUAD training cohort.

Gene	HR (95%CI)	p
ADGRD1	0.45 (0.31-0.65)	<0.001
OLFM1	0.47 (0.33-0.67)	<0.001
BTG2	0.47 (0.33-0.68)	<0.001
AC037441.1	0.47 (0.33-0.68)	<0.001
DPYSL2	0.48 (0.33-0.68)	<0.001
PRDM16	0.48 (0.33-0.69)	<0.001
PRMT8	0.48 (0.33-0.69)	<0.001
PNMA2	0.48 (0.33-0.69)	<0.001
B3GALT2	0.49 (0.34-0.7)	<0.001
DAAM2	0.5 (0.35-0.71)	<0.001
AC145124.1	0.51 (0.35-0.73)	<0.001
VWA2	0.51 (0.35-0.73)	<0.001
HSD17B6	0.51 (0.36-0.73)	<0.001
TMEM130	0.51 (0.36-0.73)	<0.001
LINC02038	0.51 (0.36-0.73)	<0.001
CTSH	0.52 (0.36-0.74)	<0.001
XCR1	0.52 (0.36-0.75)	<0.001
NT5C1A	0.52 (0.36-0.75)	<0.001
SCNN1B	0.52 (0.36-0.75)	<0.001
AC236972.3	0.53 (0.37-0.75)	<0.001
AC004947.2	0.53 (0.37-0.76)	0.001
CHRDL1	0.53 (0.37-0.76)	<0.001
FCER1A	0.53 (0.37-0.76)	<0.001
LINC00982	0.53 (0.37-0.77)	0.001
PTCSC3	0.53 (0.37-0.76)	0.001
GGT6	0.54 (0.37-0.77)	0.001
TEPP	0.54 (0.37-0.77)	0.001
AL133466.1	0.54 (0.37-0.77)	0.001
AL355974.2	0.54 (0.38-0.77)	0.001
LINC01644	0.54 (0.38-0.78)	0.001
SFTPB	0.54 (0.38-0.78)	0.001
NR0B2	0.54 (0.38-0.78)	0.001
AC007207.2	0.54 (0.38-0.78)	0.001
AC133963.1	0.55 (0.38-0.78)	0.001
NAPSA	0.55 (0.38-0.78)	0.001
SLIT3	0.55 (0.38-0.79)	0.001

ELN	0.55 (0.38-0.79)	0.001
CACNA2D2	0.55 (0.38-0.79)	0.001
KLHDC7A	0.55 (0.39-0.79)	0.001
COLCA1	0.55 (0.38-0.8)	0.001
DPPA3P2	0.56 (0.39-0.8)	0.001
SYNPR-AS1	0.56 (0.39-0.81)	0.002
EDA2R	0.56 (0.39-0.81)	0.002
AL162511.1	0.57 (0.4-0.81)	0.002
CLUL1	0.57 (0.4-0.82)	0.002
MS4A2	0.57 (0.4-0.81)	0.002
SUSD2	0.57 (0.4-0.82)	0.002
RS1	0.57 (0.4-0.82)	0.002
SFTA1P	0.57 (0.4-0.82)	0.002
FOSB	0.57 (0.4-0.82)	0.003
C14orf180	0.58 (0.4-0.82)	0.002
SEC14L6	0.58 (0.4-0.82)	0.002
LINC01819	0.58 (0.4-0.82)	0.002
ADRA2A	0.58 (0.4-0.82)	0.003
HPGDS	0.58 (0.4-0.83)	0.003
MALL	0.58 (0.4-0.83)	0.003
TMEM252	0.58 (0.4-0.83)	0.003
RCAN2	0.58 (0.41-0.83)	0.003
CADM3-AS1	0.58 (0.41-0.83)	0.003
LRRC52-AS1	0.58 (0.41-0.83)	0.003
VEGFD	0.58 (0.41-0.84)	0.003
AC091806.1	0.58 (0.41-0.83)	0.003
HMCN2	0.58 (0.41-0.83)	0.003
ABI3BP	0.58 (0.41-0.83)	0.003
CX3CR1	0.58 (0.41-0.83)	0.003
CD1E	0.59 (0.41-0.84)	0.003
AC046195.1	0.59 (0.41-0.84)	0.004
SIGLEC6	0.59 (0.41-0.84)	0.004
AC008878.3	0.59 (0.41-0.84)	0.004
GATA1	0.59 (0.41-0.84)	0.004
RNASE1	0.59 (0.41-0.84)	0.003
KCNA4	0.59 (0.41-0.84)	0.004
BRD9P2	0.59 (0.41-0.84)	0.004
AP003064.2	0.59 (0.41-0.84)	0.004
MGP	0.59 (0.41-0.84)	0.004
C1QTNF7	0.59 (0.42-0.85)	0.004
LINC00261	0.59 (0.42-0.85)	0.004
CYP4B1	0.59 (0.42-0.85)	0.004
SELENOP	0.59 (0.42-0.85)	0.004
TMEM163	0.6 (0.42-0.85)	0.004
P2RY12	0.6 (0.42-0.85)	0.004
CD1B	0.6 (0.42-0.85)	0.004
C10orf105	0.6 (0.42-0.85)	0.005
CRTAC1	0.6 (0.42-0.85)	0.005
CD207	0.6 (0.42-0.85)	0.005
NFIX	0.6 (0.42-0.86)	0.005
MAOA	0.6 (0.42-0.86)	0.005
HPSE2	0.6 (0.42-0.86)	0.005
SLC16A11	0.6 (0.42-0.86)	0.005
ACSM5	0.6 (0.42-0.86)	0.005
TMPRSS2	0.6 (0.42-0.86)	0.005
AC073862.2	0.6 (0.42-0.86)	0.005
RPS29P11	0.6 (0.42-0.86)	0.005
AC023421.1	0.6 (0.42-0.86)	0.005
CCR6	0.6 (0.42-0.86)	0.006
PPP1R1B	0.6 (0.42-0.86)	0.006
CLEC3B	0.6 (0.42-0.86)	0.005
TNS1	0.6 (0.42-0.86)	0.006
ASTN1	0.6 (0.42-0.86)	0.005
GCSAML	0.6 (0.42-0.86)	0.006
AGER	0.61 (0.42-0.87)	0.006
LINC01750	0.61 (0.42-0.86)	0.006
CHIAP2	0.61 (0.43-0.87)	0.006
SORCS1	0.61 (0.43-0.87)	0.006
ZSCAN4	0.61 (0.43-0.87)	0.007

AC012085.2	0.61 (0.43-0.87)	0.007
GRIA1	0.61 (0.43-0.87)	0.007
SCUBE2	0.61 (0.43-0.88)	0.007
C16orf89	0.61 (0.43-0.87)	0.007
KCNQ1	0.61 (0.43-0.88)	0.007
ADGRF5	0.61 (0.43-0.88)	0.007
FCGBP	0.61 (0.43-0.88)	0.007
ST6GALNAC1	0.61 (0.43-0.88)	0.007
CCL17	0.62 (0.43-0.88)	0.007
PGM5	0.62 (0.43-0.88)	0.007
IRX2	0.62 (0.43-0.88)	0.008
DNASE2B	0.62 (0.43-0.88)	0.007
LANCL3	0.62 (0.43-0.88)	0.008
RAB44	0.62 (0.43-0.88)	0.008
DLEC1	0.62 (0.43-0.88)	0.008
KCNS2	0.62 (0.43-0.88)	0.008
SLC44A4	0.62 (0.43-0.88)	0.008
CCL14	0.62 (0.43-0.88)	0.008
FO681492.1	0.62 (0.43-0.88)	0.008
CD1A	0.62 (0.43-0.88)	0.008
CHIA	0.62 (0.44-0.88)	0.008
HSD17B13	0.62 (0.43-0.88)	0.009
CYP4Z1	0.62 (0.43-0.89)	0.009
FAM189A2	0.62 (0.43-0.89)	0.009
CST5	0.62 (0.44-0.88)	0.008
CLEC9A	0.62 (0.44-0.88)	0.008
PLA2G1B	0.62 (0.44-0.89)	0.009
AL008733.1	0.62 (0.44-0.89)	0.009
HSPB7	0.62 (0.44-0.89)	0.009
EDN3	0.62 (0.44-0.89)	0.01
CLEC4F	0.62 (0.44-0.89)	0.009
AC073862.5	0.62 (0.44-0.89)	0.009
TYRP1	0.62 (0.44-0.89)	0.01
RAI2	0.62 (0.44-0.89)	0.009
SFTPD	0.62 (0.44-0.89)	0.01
ERVFRD-1	0.62 (0.44-0.89)	0.009
PLA2G3	0.63 (0.44-0.89)	0.009
KLK11	0.63 (0.44-0.9)	0.01
MUSK	0.63 (0.44-0.89)	0.01
SPARCL1	0.63 (0.44-0.9)	0.011
AC105254.2	0.63 (0.44-0.9)	0.011
STAC2	0.63 (0.44-0.9)	0.011
AC004947.1	0.63 (0.44-0.9)	0.011
ZDHHC11B	0.63 (0.44-0.9)	0.012
SLC18A2	0.63 (0.44-0.91)	0.013
RIC3	0.63 (0.44-0.9)	0.012
RMST	0.63 (0.44-0.9)	0.012
ABCC12	0.64 (0.44-0.91)	0.012
SLC34A2	0.64 (0.44-0.91)	0.013
MS4A15	0.64 (0.44-0.91)	0.013
TPPP	0.64 (0.44-0.91)	0.013
C5orf38	0.64 (0.45-0.91)	0.012
AL157895.1	0.64 (0.45-0.91)	0.013
ABCC8	0.64 (0.45-0.91)	0.014
PCDH15	0.64 (0.45-0.91)	0.013
ISLR2	0.64 (0.45-0.91)	0.014
KIAA0408	0.64 (0.45-0.91)	0.013
DRAIC	0.64 (0.45-0.91)	0.014
GDF10	0.64 (0.45-0.91)	0.013
CYP4F24P	0.64 (0.45-0.91)	0.014
ATP13A4	0.64 (0.45-0.92)	0.014
ADAMTS9-AS2	0.64 (0.45-0.91)	0.013
LINC02133	0.64 (0.45-0.92)	0.015
FHL5	0.64 (0.45-0.91)	0.014
CAVIN2	0.64 (0.45-0.91)	0.014
ADAMTS8	0.64 (0.45-0.91)	0.014
GALNT17	0.64 (0.45-0.92)	0.015
GGTLC5P	0.64 (0.45-0.92)	0.015
AC006994.2	0.64 (0.45-0.92)	0.016

PEBP4	0.65 (0.45-0.92)	0.015
ADGRB3	0.65 (0.45-0.92)	0.016
MST1L	0.65 (0.45-0.92)	0.016
AC104035.1	0.65 (0.46-0.92)	0.016
RSPO1	0.65 (0.45-0.93)	0.017
NEGR1	0.65 (0.45-0.92)	0.017
ALOX15B	0.65 (0.46-0.93)	0.017
CD1C	0.65 (0.46-0.93)	0.017
LMO3	0.65 (0.46-0.93)	0.018
FOXA2	0.65 (0.46-0.93)	0.019
PIGR	0.65 (0.46-0.93)	0.018
CAPN13	0.65 (0.46-0.93)	0.018
GFI1B	0.65 (0.46-0.93)	0.017
CNTN3	0.65 (0.46-0.93)	0.018
AL390778.2	0.65 (0.46-0.93)	0.018
RAP1GAP	0.65 (0.46-0.93)	0.019
MAB21L1	0.65 (0.46-0.93)	0.018
MFAP4	0.65 (0.46-0.93)	0.019
SFTPC	0.65 (0.46-0.93)	0.019
AC007671.1	0.65 (0.46-0.94)	0.02
FGF14	0.66 (0.46-0.93)	0.019
HMGCLL1	0.66 (0.46-0.93)	0.019
FOS	0.66 (0.46-0.94)	0.02
MAP6	0.66 (0.46-0.93)	0.019
LHFPL3-AS2	0.66 (0.46-0.93)	0.019
AC010998.1	0.66 (0.46-0.93)	0.019
ZNF366	0.66 (0.46-0.94)	0.021
MIR663AHG	0.66 (0.46-0.94)	0.02
ADAMTS7P3	0.66 (0.46-0.93)	0.019
BAAT	0.66 (0.46-0.94)	0.02
RN7SL8P	0.66 (0.46-0.94)	0.021
SHE	0.66 (0.46-0.94)	0.022
CHRM2	0.66 (0.46-0.94)	0.02
PLA2G12B	0.66 (0.46-0.94)	0.02
LRRC52	0.66 (0.46-0.94)	0.022
MIR4697HG	0.66 (0.46-0.95)	0.024
SLC13A2	0.66 (0.46-0.94)	0.023
SLC46A2	0.66 (0.47-0.94)	0.022
CPA3	0.66 (0.46-0.94)	0.023
INMT	0.66 (0.46-0.94)	0.022
WSCD2	0.66 (0.46-0.94)	0.023
GGTLC1	0.66 (0.47-0.94)	0.022
NR3C2	0.66 (0.46-0.94)	0.023
RASGRF1	0.66 (0.46-0.95)	0.024
ENAM	0.66 (0.47-0.94)	0.022
MYH11	0.66 (0.46-0.95)	0.024
KCND3	0.66 (0.47-0.95)	0.024
SEC14L3	0.66 (0.47-0.94)	0.023
CDKL2	0.66 (0.47-0.95)	0.023
SCN2B	0.66 (0.47-0.95)	0.024
SHISA2	0.66 (0.47-0.94)	0.023
RN7SKP51	0.66 (0.47-0.95)	0.024
CYBRD1	0.66 (0.47-0.95)	0.024
AC005165.1	0.66 (0.47-0.95)	0.024
ELN-AS1	0.66 (0.47-0.95)	0.024
LPAL2	0.67 (0.47-0.95)	0.026
RHOBTB2	0.67 (0.47-0.95)	0.026
C2orf40	0.67 (0.47-0.95)	0.024
MIR3189	0.67 (0.47-0.96)	0.027
ABCA3	0.67 (0.47-0.96)	0.028
AL445493.3	0.67 (0.47-0.96)	0.027
ABCA8	0.67 (0.47-0.95)	0.026
PENK	0.67 (0.47-0.95)	0.025
GAS2L2	0.67 (0.47-0.96)	0.029
AL135999.3	0.67 (0.47-0.95)	0.026
HLF	0.67 (0.47-0.96)	0.028
SLC8A3	0.67 (0.47-0.96)	0.027
KLF15	0.67 (0.47-0.96)	0.028
PARM1	0.67 (0.47-0.96)	0.028

PIFO	0.67 (0.47-0.96)	0.029
TMEM132E	0.67 (0.47-0.96)	0.029
PCDH20	0.67 (0.47-0.96)	0.031
HAGLR	0.68 (0.47-0.96)	0.03
C4A	0.68 (0.47-0.96)	0.031
LCN6	0.68 (0.47-0.96)	0.03
AC005884.1	0.68 (0.48-0.96)	0.029
FGF10	0.68 (0.47-0.96)	0.031
CPAMD8	0.68 (0.47-0.96)	0.03
ADGRF5P2	0.68 (0.47-0.96)	0.031
MYOM2	0.68 (0.48-0.96)	0.03
AC093523.1	0.68 (0.47-0.97)	0.032
SCTR	0.68 (0.48-0.97)	0.033
COLEC12	0.68 (0.48-0.97)	0.032
SIGLEC17P	0.68 (0.48-0.97)	0.033
ADRB3	0.68 (0.48-0.97)	0.031
LINC01936	0.68 (0.48-0.97)	0.033
CYP2A6	0.68 (0.48-0.97)	0.033
C1orf116	0.68 (0.48-0.97)	0.033
LINC02568	0.68 (0.48-0.97)	0.034
FOLR1	0.68 (0.48-0.97)	0.035
PHACTR1	0.68 (0.48-0.97)	0.034
LINC02519	0.68 (0.48-0.97)	0.033
FPGT-TNNI3K	0.68 (0.48-0.98)	0.036
GPIHBP1	0.68 (0.48-0.97)	0.035
CXCL17	0.68 (0.48-0.98)	0.036
AP004608.1	0.68 (0.48-0.98)	0.037
F11	0.69 (0.48-0.98)	0.037
CA10	0.69 (0.48-0.98)	0.037
PI16	0.69 (0.48-0.97)	0.035
PRIMA1	0.69 (0.48-0.98)	0.036
MAGEE2	0.69 (0.48-0.98)	0.037
ECEL1P2	0.69 (0.48-0.98)	0.036
AC078925.4	0.69 (0.48-0.98)	0.037
CYP2B7P	0.69 (0.48-0.98)	0.038
PTGDS	0.69 (0.48-0.98)	0.038
AC022164.1	0.69 (0.48-0.98)	0.039
AP003481.1	0.69 (0.48-0.98)	0.037
DNAI2	0.69 (0.48-0.98)	0.039
TPSB2	0.69 (0.48-0.98)	0.04
VIPR1	0.69 (0.48-0.99)	0.042
SLC14A1	0.69 (0.48-0.98)	0.04
AC092071.1	0.69 (0.48-0.99)	0.044
BMX	0.69 (0.49-0.99)	0.042
TBX4	0.69 (0.49-0.99)	0.042
LHFPL3	0.69 (0.49-0.99)	0.042
AC096531.2	0.69 (0.49-0.99)	0.042
NR1H4	0.69 (0.49-0.99)	0.042
ITGA9	0.69 (0.49-0.99)	0.043
SERPIND1	0.7 (0.49-0.99)	0.043
PRODH	0.7 (0.49-0.99)	0.045
CFAP221	0.7 (0.49-0.99)	0.042
SLC22A31	0.7 (0.49-0.99)	0.045
IRX1	0.7 (0.49-0.99)	0.046
ADGRD1-AS1	0.7 (0.49-0.99)	0.045
SCGB3A2	0.7 (0.49-0.99)	0.046
SHH	0.7 (0.49-1)	0.047
MIR27A	0.7 (0.49-1)	0.047
INMT-MINDY4	0.7 (0.49-0.99)	0.046
OTC	0.7 (0.49-1)	0.047
ART4	0.7 (0.49-0.99)	0.046
PRELP	0.7 (0.49-1)	0.048
AC093772.1	0.7 (0.49-0.99)	0.046
RPL13AP17	0.7 (0.49-1)	0.049
BPIFB2	0.7 (0.49-1)	0.048
AC090092.1	0.7 (0.49-1)	0.049
AC112907.2	1.42 (1-2.03)	0.049
GUCA1A	1.43 (1-2.04)	0.049
CCDC150	1.43 (1.01-2.04)	0.046

STIL	1.44 (1.01-2.04)	0.045
HIST1H1E	1.44 (1.01-2.05)	0.045
AL138789.1	1.44 (1.01-2.04)	0.045
NCAPH	1.44 (1.01-2.04)	0.043
KIF2C	1.44 (1.01-2.04)	0.043
TWIST1	1.45 (1.01-2.06)	0.042
RAB3B	1.45 (1.01-2.07)	0.041
TYMSOS	1.45 (1.02-2.07)	0.039
RACGAP1	1.46 (1.02-2.07)	0.037
ADGRF4	1.46 (1.02-2.09)	0.039
AP000526.1	1.46 (1.02-2.08)	0.036
FDPSP8	1.47 (1.03-2.09)	0.036
FAM83A-AS1	1.47 (1.03-2.09)	0.034
E2F1	1.47 (1.03-2.09)	0.033
AC005042.1	1.47 (1.03-2.1)	0.033
ASF1B	1.47 (1.03-2.1)	0.032
C1QL1	1.48 (1.04-2.1)	0.031
CCNE2	1.48 (1.04-2.1)	0.031
EPHA6	1.48 (1.04-2.11)	0.031
RAD54L	1.48 (1.04-2.1)	0.03
PARPBP	1.48 (1.04-2.11)	0.03
AC012073.1	1.48 (1.04-2.11)	0.029
UHRF1	1.48 (1.04-2.11)	0.029
DNA2	1.48 (1.04-2.11)	0.028
TRAIP	1.48 (1.04-2.12)	0.029
RCOR2	1.49 (1.04-2.12)	0.029
HOXA13	1.49 (1.04-2.12)	0.029
CDC20	1.49 (1.05-2.12)	0.027
RECQL4	1.49 (1.05-2.12)	0.027
GPR19	1.49 (1.04-2.12)	0.028
ACTL8	1.49 (1.05-2.13)	0.026
RFC4	1.49 (1.05-2.13)	0.026
HIST1H2AM	1.49 (1.05-2.13)	0.027
LINC01116	1.5 (1.04-2.14)	0.028
COL11A1	1.5 (1.05-2.14)	0.027
HIST2H2AB	1.5 (1.05-2.14)	0.026
NR5A1	1.5 (1.05-2.14)	0.025
FGF5	1.5 (1.05-2.15)	0.026
WT1-AS	1.51 (1.06-2.15)	0.024
HOXA11	1.51 (1.06-2.16)	0.024
KIF15	1.51 (1.06-2.15)	0.022
CKAP2L	1.51 (1.06-2.15)	0.021
KIF4A	1.51 (1.07-2.15)	0.021
OR51E1	1.51 (1.06-2.17)	0.023
IL36RN	1.52 (1.07-2.17)	0.02
EME1	1.52 (1.07-2.17)	0.019
LINC01213	1.52 (1.07-2.17)	0.019
MYBL2	1.53 (1.07-2.18)	0.019
SGO1	1.53 (1.07-2.17)	0.018
MKRN9P	1.53 (1.07-2.18)	0.019
TERT	1.53 (1.08-2.18)	0.018
AC012213.1	1.53 (1.07-2.18)	0.018
MCM4	1.53 (1.08-2.18)	0.017
ZWINT	1.53 (1.08-2.18)	0.017
MUC5B	1.55 (1.09-2.2)	0.015
CDC25A	1.55 (1.09-2.21)	0.015
SHOC1	1.55 (1.08-2.21)	0.016
AC112777.1	1.55 (1.09-2.21)	0.015
KIF18A	1.55 (1.08-2.22)	0.016
NPM1P9	1.55 (1.08-2.23)	0.017
BIRC5	1.56 (1.09-2.22)	0.015
LINC02323	1.56 (1.09-2.22)	0.015
KPNA2	1.56 (1.09-2.22)	0.014
WDHD1	1.56 (1.09-2.22)	0.014
PITX1	1.56 (1.09-2.22)	0.014
AC027627.1	1.56 (1.1-2.22)	0.014
LY6K	1.56 (1.09-2.22)	0.014
HPDL	1.56 (1.09-2.23)	0.015
HIST1H2BJ	1.56 (1.09-2.24)	0.015

NCAPG	1.57 (1.1-2.23)	0.013
C5orf34	1.57 (1.1-2.25)	0.012
DLX2	1.58 (1.1-2.25)	0.012
MYEOV	1.58 (1.1-2.26)	0.013
CLSPN	1.58 (1.11-2.25)	0.011
STEAP2-AS1	1.58 (1.11-2.26)	0.011
DEPDC1	1.58 (1.11-2.26)	0.011
SGO2	1.58 (1.11-2.26)	0.011
UBE2T	1.59 (1.11-2.26)	0.011
FOSL1	1.59 (1.11-2.27)	0.011
ESPL1	1.59 (1.12-2.26)	0.01
BRCA1	1.59 (1.12-2.27)	0.01
DTL	1.59 (1.12-2.27)	0.01
SEPT14P12	1.59 (1.12-2.27)	0.01
RAD54B	1.6 (1.12-2.29)	0.009
ORC1	1.6 (1.12-2.28)	0.009
EIF4EBP1	1.61 (1.12-2.3)	0.01
CDC45	1.61 (1.13-2.29)	0.008
SBSN	1.61 (1.13-2.29)	0.009
AC006329.1	1.61 (1.13-2.3)	0.009
EREG	1.61 (1.13-2.3)	0.008
ORC6	1.62 (1.13-2.31)	0.008
RRM2	1.62 (1.13-2.31)	0.008
CENPA	1.62 (1.13-2.31)	0.008
RIMS2	1.62 (1.13-2.31)	0.008
POLR3G	1.62 (1.13-2.32)	0.009
DSCC1	1.62 (1.14-2.31)	0.008
MND1	1.62 (1.13-2.32)	0.008
AURKA	1.62 (1.14-2.31)	0.008
CENPF	1.63 (1.14-2.32)	0.007
AC068228.1	1.63 (1.14-2.33)	0.007
CTSV	1.63 (1.14-2.33)	0.007
GINS4	1.63 (1.15-2.33)	0.007
BUB1	1.64 (1.15-2.33)	0.007
NDC80	1.64 (1.15-2.33)	0.007
UBE2S	1.64 (1.14-2.34)	0.007
TTK	1.64 (1.15-2.33)	0.006
PSRC1	1.64 (1.15-2.34)	0.007
SPC24	1.64 (1.15-2.34)	0.007
DDIAS	1.64 (1.15-2.34)	0.006
CSAG1	1.64 (1.15-2.35)	0.007
RAD51AP1	1.64 (1.15-2.34)	0.006
BRIP1	1.65 (1.15-2.35)	0.006
AP002478.1	1.65 (1.15-2.35)	0.006
SPC25	1.65 (1.16-2.35)	0.006
HIST1H2BO	1.65 (1.15-2.35)	0.006
ESCO2	1.65 (1.16-2.36)	0.006
UBE2C	1.65 (1.16-2.36)	0.006
TEDC2	1.66 (1.16-2.36)	0.006
HJURP	1.67 (1.17-2.38)	0.005
CENPU	1.67 (1.17-2.39)	0.005
FAM72A	1.67 (1.17-2.39)	0.004
CDKN3	1.67 (1.17-2.39)	0.005
HIST1H3F	1.68 (1.16-2.41)	0.005
PCMTD1P3	1.68 (1.18-2.4)	0.004
PRR11	1.68 (1.18-2.4)	0.004
FAM111B	1.68 (1.18-2.41)	0.004
MTBP	1.69 (1.18-2.4)	0.004
AC073585.1	1.69 (1.18-2.41)	0.004
PBK	1.69 (1.18-2.41)	0.004
CEP55	1.7 (1.19-2.42)	0.004
PLK1	1.7 (1.19-2.42)	0.003
SKA1	1.7 (1.19-2.42)	0.004
GBX2	1.7 (1.19-2.43)	0.004
HIST1H4D	1.7 (1.18-2.44)	0.004
AUNIP	1.7 (1.19-2.44)	0.004
RGS20	1.7 (1.19-2.44)	0.003
KIF18B	1.71 (1.2-2.43)	0.003
SHCBP1	1.71 (1.19-2.45)	0.003

POC1A	1.71 (1.2-2.44)	0.003
TYMS	1.71 (1.2-2.44)	0.003
POLQ	1.72 (1.2-2.44)	0.003
ASPM	1.72 (1.21-2.45)	0.003
NEK2	1.72 (1.2-2.45)	0.003
FAM72C	1.72 (1.2-2.46)	0.003
CENPW	1.72 (1.2-2.46)	0.003
MCM10	1.72 (1.21-2.46)	0.003
HIST1H2AL	1.72 (1.2-2.47)	0.003
TROAP	1.72 (1.21-2.46)	0.003
CHEK1	1.72 (1.21-2.46)	0.003
DLGAP5	1.73 (1.21-2.46)	0.003
PCLAF	1.73 (1.21-2.47)	0.003
ITGB1-DT	1.73 (1.2-2.48)	0.003
TICRR	1.73 (1.21-2.46)	0.002
KIF11	1.73 (1.21-2.47)	0.002
PKMYT1	1.73 (1.21-2.47)	0.002
RAD51	1.73 (1.21-2.48)	0.003
XRCC2	1.74 (1.22-2.47)	0.002
ATP5MC1P4	1.74 (1.21-2.49)	0.003
CCNE1	1.74 (1.22-2.49)	0.002
OIP5	1.74 (1.22-2.49)	0.002
CDCA5	1.75 (1.22-2.49)	0.002
KIFC1	1.75 (1.22-2.49)	0.002
AURKB	1.75 (1.22-2.5)	0.002
AC016877.3	1.75 (1.22-2.5)	0.002
CDK1	1.75 (1.22-2.5)	0.002
FBXO43	1.75 (1.23-2.5)	0.002
TPX2	1.76 (1.23-2.51)	0.002
DUSP5P1	1.76 (1.24-2.51)	0.002
ECE2	1.76 (1.23-2.54)	0.002
NUSAP1	1.77 (1.24-2.52)	0.002
HIST1H2BF	1.77 (1.23-2.54)	0.002
AC016205.1	1.77 (1.24-2.53)	0.002
KIF20A	1.77 (1.24-2.54)	0.002
MELK	1.78 (1.24-2.54)	0.002
MAD2L1	1.78 (1.24-2.55)	0.002
KNL1	1.78 (1.25-2.54)	0.001
ERCC6L	1.79 (1.25-2.56)	0.001
AC099850.3	1.79 (1.25-2.57)	0.001
POLE2	1.79 (1.26-2.56)	0.001
CDCA2	1.8 (1.26-2.56)	0.001
ACRV1	1.8 (1.26-2.58)	0.001
UBE2SP1	1.8 (1.26-2.58)	0.001
E2F7	1.8 (1.26-2.58)	0.001
DEPDC1B	1.81 (1.26-2.59)	0.001
NUF2	1.81 (1.27-2.59)	0.001
ARHGEF39	1.81 (1.27-2.6)	0.001
PLK4	1.81 (1.27-2.59)	0.001
CENPE	1.82 (1.27-2.6)	0.001
GAL	1.82 (1.27-2.62)	0.001
SAPCD2	1.82 (1.27-2.61)	0.001
FAM83D	1.83 (1.27-2.62)	0.001
EXO1	1.83 (1.28-2.61)	0.001
CENPK	1.83 (1.28-2.63)	0.001
CCNB1	1.84 (1.28-2.63)	0.001
SPAG5	1.85 (1.29-2.64)	0.001
FAM72D	1.85 (1.29-2.65)	0.001
PIMREG	1.85 (1.29-2.65)	0.001
SKA3	1.85 (1.3-2.65)	0.001
CDCA3	1.86 (1.3-2.66)	0.001
PTTG1	1.87 (1.3-2.69)	0.001
CCNB2	1.87 (1.31-2.68)	0.001
HASPIN	1.88 (1.31-2.68)	0.001
FAM83A	1.88 (1.31-2.7)	0.001
GTSE1	1.88 (1.32-2.69)	0.001
GREB1L	1.89 (1.32-2.71)	0.001
HMMR	1.9 (1.33-2.72)	<0.001
CENPM	1.9 (1.32-2.73)	0.001

OR2B6	1.91 (1.34-2.73)	<0.001
NEIL3	1.92 (1.33-2.77)	0.001
PRC1	1.92 (1.34-2.75)	<0.001
STRIP2	1.92 (1.34-2.76)	<0.001
ARHGAP11A	1.93 (1.35-2.76)	<0.001
FOXM1	1.93 (1.35-2.76)	<0.001
CCNA2	1.94 (1.35-2.77)	<0.001
FAM72B	1.94 (1.35-2.78)	<0.001
ANLN	1.94 (1.36-2.78)	<0.001
BUB1B	1.95 (1.36-2.79)	<0.001
TK1	1.96 (1.36-2.81)	<0.001
KIF23	1.96 (1.37-2.8)	<0.001
VGF	1.96 (1.37-2.82)	<0.001
DIAPH3	1.98 (1.38-2.83)	<0.001
MTFR2	1.99 (1.39-2.85)	<0.001
MKI67	1.99 (1.39-2.85)	<0.001
HELLS	2 (1.4-2.85)	<0.001
TESMIN	2.02 (1.41-2.9)	<0.001
AC087588.2	2.04 (1.42-2.93)	<0.001
TRIP13	2.05 (1.43-2.95)	<0.001
AL031777.1	2.06 (1.42-2.98)	<0.001
CDC25C	2.07 (1.44-2.98)	<0.001
C17orf53	2.09 (1.46-2.99)	<0.001
KIF14	2.19 (1.53-3.14)	<0.001
DNMT3B	2.23 (1.56-3.21)	<0.001

Table S4. Survival-related DEGs in TCGA-LUAD training cohort.

Gene	Coefficients
HOXA11	0
ABCC8	0
SLC13A2	0
CACNA2D2	0
DLEC1	0
NFIX	0
RHOBTB2	0
ANLN	0
BRCA1	0
NR1H4	0
DEPDC1	0
HSD17B6	0
MUSK	0
DEPDC1B	0
MYOM2	0
OTC	0
LMO3	0
GUCA1A	0
ELN	0
RAD51	0
POLQ	0
KCNQ1	0
RASGRF1	0
COL11A1	0
MCM10	0
ASPM	0
PRR11	0
PITX1	0
ADGRF5	0
GAL	0
FGF10	0
ST6GALNAC1	0
TRIP13	0
CYBRD1	0
HMMR	0
WSCD2	0
GTSE1	0
SPAG5	0
RAP1GAP	0

UBE2T	0
BPIFB2	0
CLUL1	0
TNS1	0
EPHA6	0
SCTR	0
NDC80	0
ORC1	0
RAD54L	0
AURKA	0
DNMT3B	0
TPX2	0
F11	0
BIRC5	0
KIF4A	0
ORC6	0
CLSPN	0
DPYSL2	0
CDC45	0
CRTAC1	0
SERPIND1	0
SUSD2	0
SEC14L3	0
PRODH	0
PLA2G3	0
CENPM	0
POLE2	0
CDKN3	0
SLC8A3	0
MYBL2	0
E2F1	0
FAM83D	0
CHRDL1	0
BMX	0
RS1	0
GATA1	0
FGF14	0
CCL17	0
CTSH	0
OIP5	0
MCM4	0
ASF1B	0
CCNE1	0
SIGLEC6	0
HOXA13	0
TYRP1	0
PTGDS	0
SORCS1	0
UBE2S	0
HLF	0
NEIL3	0
NCAPG	0
FOLR1	0
FOXM1	0
PRMT8	0
RAD51AP1	0
ART4	0
MGP	0
ADGRD1	0
CDCA3	0
PHACTR1	0
FHL5	0
CCR6	-0.032977722
TTK	0
KIF20A	0
POLR3G	0
CNTN3	0
VIPR1	0
CENPA	0

DLX2	0
CD207	0
NT5C1A	0
ACTL8	0
CDC20	0
NEK2	0
CENPF	0
MUC5B	0
KIF14	0
C2orf40	0
SLC46A2	0
HELLS	0
TBX4	0
NCAPH	0
MND1	0
KIF18A	0
TWIST1	0
ZWINT	0
CENPK	0
STIL	0
HJURP	0
EDN3	0
HIST1H2BJ	0
OR2B6	0
EREG	0
C17orf53	0
FOSB	0
FOXA2	0
DLGAP5	0
ATP13A4	0
AUNIP	0
PKMYT1	0
VGF	0
STRIP2	0
PIMREG	0
RNASE1	0
SGO1	0
OLFM1	0
EDA2R	0
C1QL1	0
NAPSA	0
PPP1R1B	0
RAI2	0
NR0B2	0
ENAM	0
TESMIN	0.002511291
MYH11	0
SFTPD	0
PEBP4	0
CCNB1	0
CHIA	0
PSRC1	0
ADAMTS8	0
ACRV1	0
FAM189A2	0
ADGRB3	0
TROAP	0
ESPL1	0
BRIP1	0
IL36RN	0
BAAT	0
NR5A1	0
CTSV	0
DSCC1	0
ARHGEF39	0
NUSAP1	0
KIF23	0
KNL1	0
DNASE2B	0

KIF11	0
CEP55	0
PLA2G12B	0
DNA2	0
FGF5	0
CDKL2	0
CENPE	0
DIAPH3	0
TICRR	0
ABCC12	-0.009507364
ABCA8	0
GREB1L	0
SLC14A1	0
STAC2	0
PRDM16	0
PLK4	0
KIF2C	0
CYP4B1	0
NUF2	0
DTL	0
MALL	0
CCDC150	0
ITGA9	0
ECE2	0
CCNA2	0
DAAM2	0
HMGCLL1	0
MTFR2	0
CDCA5	0
LANCL3	0
RGS20	0
GINS4	0
FAM83A	0.026787288
HMCN2	0
MKI67	0
GGTLC1	0
MS4A2	0
CHEK1	0
SCN2B	0
PCDH15	0
ADRA2A	0
NR3C2	0
CENPU	0
ASTN1	0
TMEM163	0
SPC25	0
SPARCL1	0
CLEC4F	0
ADGRF4	0
C16orf89	0
ABI3BP	0
PGM5	0
SKA1	0
EME1	0
CA10	0
GRIA1	0
KCNS2	0
FBXO43	0
BUB1B	0
CCNB2	0
SLC34A2	0
COLEC12	0
CDC25C	0
CD1A	0
CD1C	0
CD1B	0
CD1E	0
BTG2	0
TEPP	0

CPAMD8	0
LY6K	0
RECQL4	0
RACGAP1	0
SPC24	0
TEDC2	0
B3GALT2	-0.039551547
LRRC52	0
PIGR	0
CAPN13	0
CFAP221	0
HPGDS	0
C1QTNF7	0
LINC01116	0
SGO2	0
CPA3	0
KIF15	0
CLEC3B	0
KLF15	0
RFC4	0
CDC25A	0
POC1A	0
MAD2L1	0
SCGB3A2	0
TERT	0
PI16	0
PTTG1	0
SHH	0
SHOC1	0
VEGFD	0
MELK	0
SKA3	0
DDIAS	0
SLC18A2	0
GFI1B	0
VWA2	0
E2F7	0
RIC3	0
TMEM130	0
MFAP4	0
PCLAF	0
PLK1	0
MS4A15	0
ISLR2	0
GGT6	0
KLK11	0
RCOR2	0
TK1	0
ABCA3	0
PBK	0
HIST1H1E	0
CX3CR1	0
SCNN1B	0
SFTPC	0
CAVIN2	0
GBX2	0
SFTPB	0
PARM1	0
RAB3B	0
RSPO1	0
GCSAML	0
SHE	0
P2RY12	0
CKAP2L	0
BUB1	0
CDK1	0
FOS	0
CST5	0
HSD17B13	0

IRX1	0
IRX2	0
PLA2G1B	0
SIGLEC17P	0
SHCBP1	0
ESCO2	0
TPPP	0
KCND3	0
MAP6	0
DNAI2	0
RRM2	0
MTBP	0
C5orf34	0
NEGR1	0
RCAN2	0
MYEOV	0
HPSE2	0
XCR1	0
HSPB7	0
PIFO	0
SLC16A11	0
EXO1	0
UBE2C	0
CCNE2	0
SCUBE2	0
FOSL1	0.017665852
PRIMA1	0
RIMS2	0
TYMS	0
TYMSOS	0
LINC00982	0
HASPIN	0
ZNF366	0
AURKB	0
KLHDC7A	0
ALOX15B	0
FCER1A	0
ZSCAN4	0
MAB21L1	0
SHISA2	0
OR51E1	0
CHRM2	0
PENK	0
TMEM132E	0
TMEM252	0
KCNA4	0
KPNA2	0
C1orf116	0
GPR19	0
WT1-AS	0
ACSM5	0
TRAIP	0
DUSP5P1	0.012717299
TMPRSS2	0
HIST2H2AB	0
SLIT3	0
C14orf180	0
CDCA2	0
GALNT17	0
PARPBP	0
CYP4Z1	0
KIF18B	0
SAPCD2	0
C5orf38	0
HPDL	0
MAGEE2	0
MST1L	0
ERCC6L	0
LHFPL3	0

EIF4EBP1	0
FAM72B	0
ADRB3	0
PRELP	0
DPPA3P2	-0.042453178
SBSN	0
FAM111B	0
MAOA	0
KIAA0408	0
CXCL17	0
COLCA1	0
FAM72A	0
XRCC2	0
TPSB2	0
RAD54B	0
CLEC9A	0
WDHD1	0
ARHGAP11A	0
PRC1	0
CSAG1	0
CENPW	0
CHIAP2	0
AGER	0
SLC44A4	0
ADGRF5P2	0
FAM83A-AS1	0
ZDHHC11B	0
MIR27A	0
LPAL2	0
SEC14L6	-0.000163888
C10orf105	0
FAM72D	0
AL031777.1	0.00083617
LINC01644	0
RN7SKP51	0
AC005165.1	0
HAGLR	0
FDPSP8	0
LHFPL3-AS2	0
SFTA1P	0
CADM3-AS1	0
LINC02519	0
AL008733.1	0
MIR663AHG	0
AC010998.1	0
AC005042.1	0
ATP5MC1P4	0.010376482
STEAP2-AS1	0
LINC02038	0
ITGB1-DT	0
NPM1P9	0
RPL13AP17	0
LINC01750	0
LINC01819	0
AC112907.2	0
ELN-AS1	0
AC006329.1	0
AL138789.1	0
AC004947.1	0
AL390778.2	0
UBE2SP1	0
AL157895.1	0
PCMTD1P3	0
SEPT14P12	0
LINC01936	0
AL133466.1	0
AC091806.1	0
AC096531.2	0
LRRC52-AS1	0

KIFC1	0
PNMA2	0
SYNPR-AS1	0
INMT	0
ADAMTS9-AS2	0
RPS29P11	0
RN7SL8P	0
ECEL1P2	0
ERVFRD-1	0
LINC01213	0
C4A	0
DRAIC	0
AC093772.1	0
AC133963.1	-0.02884125
AC037441.1	-0.028155115
AC027627.1	0
AC093523.1	0
BRD9P2	-0.030238138
SELENOP	0
AC068228.1	0
AC046195.1	0
AC012213.1	0
INMT-MINDY4	0
AC090092.1	0
AP003064.2	0
AC145124.1	0
AP004608.1	0
RAB44	0
AC073585.1	0
RMST	0
CYP2A6	0
ADGRD1-AS1	0
CYP2B7P	0
AC112777.1	0
AC007207.2	0
MKRN9P	0
AC012085.2	0
AC104035.1	0
AL162511.1	0
FPGT-TNNI3K	0
PTCSC3	0
LINC02323	0
LINC02568	0
SLC22A31	0
LINC00261	0
AC022164.1	0
ADAMTS7P3	0
LINC02133	0
FAM72C	0
MIR3189	0
AC099850.3	0
AP002478.1	0.000310756
GDF10	0
AC023421.1	0
LCN6	0
AC005884.1	0
AC016205.1	0
CYP4F24P	0
AC008878.3	0
AC092071.1	0
GAS2L2	0
AP000526.1	0
AC105254.2	0
AC016877.3	0
AC012073.1	0
AP003481.1	0
AC236972.3	0
AL135999.3	0
AC007671.1	0

HIST1H2BO	0
AC087588.2	0.004673826
AL445493.3	0
FCGBP	0
AL355974.2	0
UHRF1	0
GGTLC5P	0
CCL14	0
HIST1H2AL	0
HIST1H4D	0
HIST1H2BF	0
GPIHBP1	0
FO681492.1	0
HIST1H3F	0
HIST1H2AM	0
AC006994.2	0
AC073862.2	0
AC078925.4	0
PCDH20	0
AC073862.5	0
MIR4697HG	0
AC004947.2	-0.007125174

Table S5. The coefficients of each survival-related DEG by LASSO analysis.
